# The Mechanism of LTX^N4C^-Induced Ca^2+^ Influx Involves Latrophilin-Mediated Activation of Ca_v_2.x Channels

**DOI:** 10.3390/ijms262211200

**Published:** 2025-11-19

**Authors:** Jennifer K. Blackburn, John-Paul Silva, Evelina Petitto, Dietmar Cholewa, Elizaveta Fasler-Kan, Kirill E. Volynski, Yuri A. Ushkaryov

**Affiliations:** 1 Medway School of Pharmacy, University of Kent, Chatham ME4 4TB, UK; jennifer.blackburn@yale.edu (J.K.B.); evelina.petitto@ashfiedmedcomms.com (E.P.); 2 Department of Biological Sciences, Imperial College London, London SW7 2AZ, UKk.volynski@ucl.ac.uk (K.E.V.); 3 Department of Pediatric Surgery, Inselspital Bern, University of Bern, CH-3010 Bern, Switzerland; dietmar.cholewa@insel.ch (D.C.); elizaveta.fasler@insel.ch (E.F.-K.)

**Keywords:** latrophilin, ADGRL1, LTX^N4C^, store-operated calcium entry, voltage-gated calcium channels, fluorescent microscopy, Fluo-4, GCaMP

## Abstract

Store-operated Ca^2+^ entry (SOCE) is a key regulator of cytosolic Ca^2+^ (Ca^2+^_cyt_). Presynaptic SOCE can be activated by ligands like α-latrotoxin, which acts through the presynaptic G-protein-coupled receptor latrophilin-1 (LPHN1), inducing Ca^2+^ influx and neurotransmitter release. To understand how SOCE-associated proteins contribute to LPHN1 signaling in neurons, we used mouse neuroblastoma NB2a cells as a genetically tractable neuronal model. The cells were stably transfected with exogenous LPHN1 or its non-signaling mutant and stimulated with the non-pore-forming α-latrotoxin mutant LTX^N4C^, a known trigger of neurotransmitter release. LPHN1 expression increased the proportion of neuron-like cells and upregulated the voltage-gated Ca^2+^ channels Ca_v_1.2 and Ca_v_2.1. LPHN1 stimulation by LTX^N4C^ induced a small Ca^2+^ release sensitive to thapsigargin, and a strong, gradual influx of Ca^2+^, which was insensitive to thapsigargin. Single-cell imaging revealed that this influx consisted of desynchronized high-amplitude Ca^2+^ oscillations in individual cells. This response was reduced by Orai2 knockdown and completely blocked by the Ca_v_2.1/2.2 inhibitor ω-conotoxin MVIIC. We conclude that LPHN1 activation by LTX^N4C^ primes Ca^2+^ stores and induces the opening of Ca_v_2.1/2.2 channels. These channels mediate an initial Ca^2+^ influx that triggers Ca^2+^-induced Ca^2+^ release and SOCE. This mechanism, elucidated in model cells, can explain how LTX^N4C^ stimulates neurotransmitter release.

## 1. Introduction

Neurotransmitter release at presynaptic terminals is orchestrated by sophisticated Ca^2+^ dynamics, where influx through voltage-gated Ca^2+^ channels (VGCCs) creates a Ca^2+^ transient, which provides the immediate and essential trigger for synchronous, phasic release of neurotransmitters within sub-millisecond timescales [1,2,3]. While being mainly driven by the canonical influx through VGCCs, this Ca^2+^ transient is modified by the coordinated activity of multiple other sources of Ca^2+^ [4].

In addition to vesicle fusion, the initial Ca^2+^ influx through VGCCs triggers Ca^2+^-induced Ca^2+^ release (CICR) from the presynaptic endoplasmic reticulum (ER) stores via ryanodine receptors (RyRs) [5,6]. This secondary release sustains and amplifies the Ca^2+^ transients during repetitive stimulation [7,8]; modulates asynchronous release [9,10], regulates short-term plasticity [11,12], and supports spontaneous release (minis) [13].

Recent advances have identified store-operated Ca^2+^ entry (SOCE) as another crucial regulator of presynaptic Ca^2+^ homeostasis [14,15], particularly during sustained neuronal activity [16]. The SOCE mechanism is initiated when Ca^2+^ is released from the ER Ca^2+^ stores [17]. Store depletion triggers the oligomerization of ER-resident stromal interaction molecules (STIM1/2), which then translocate to ER-plasma membrane junctions, where they activate Orai1 channels in the plasma membrane and thus induce highly selective influx of extracellular Ca^2+^ (Ca^2+^_e_). In neurons, the ER extends into axons and nerve terminals, allowing STIM proteins to mediate presynaptic SOCE [18]. Apart from its role in maintaining the ER Ca^2+^ stores, presynaptic SOCE modulates fundamental synaptic properties including vesicle release probability and post-tetanic potentiation [5,19,20], with emerging evidence indicating the influence of presynaptic SOCE on the availability of readily releasable vesicles, which implies a role in vesicle priming and spontaneous release [19,21].

The activation of SOCE is also dynamically controlled by G protein-coupled receptors (GPCRs), which serve as critical transducers of extracellular signals into intracellular Ca^2+^ responses [22]. Canonical GPCR signaling through Gαq/11 subunits stimulates phospholipase Cβ (PLCβ), which hydrolyses phosphatidylinositol 4,5-bisphosphate (PIP_2_), generating inositol 1,4,5-trisphosphate (IP_3_) and diacylglycerol (DAG). IP_3_ activates the ER-resident IP_3_ receptors, which release Ca^2+^ and thereby initiate SOCE [23]. DAG, acting via protein kinase C (PKC) or directly, modulates (primarily activates) many types of Ca^2+^ channels, including some VGCCs, Orai channels, TRPC channels, IP_3_ receptors, and RyRs. Alternative pathways may engage SOCE through PLC-independent mechanisms [24], potentially involving direct STIM recruitment or lipid-mediated signaling [25,26]. These diverse activation mechanisms position GPCRs as central regulators of presynaptic SOCE, capable of integrating Ca^2+^ signaling pathways with synaptic activity.

To investigate the role of extracellular ligands in GPCR-mediated control of SOCE, we selected the mouse neuroblastoma Neuro-2a cell line (NB) as an experimentally tractable model system that recapitulates key features of neuronal Ca^2+^ regulation [27]. When differentiated through serum withdrawal or cAMP elevation, NB cells undergo morphological and functional transformation, extending neurite-like processes and generating specialized compartments resembling presynaptic terminals, which enables detailed investigation of Ca^2+^ signals with spatial organization similar to that in neurons. Upon differentiation, NB cells express neuronal markers including synaptophysin and MAP2 [28,29,30], while maintaining endogenous expression of critical Ca^2+^ handling proteins such as STIM/Orai, RyRs, and Ca^2+^ ATPases [31,32,33,34]. By contrast, they do not express latrophilin 1 (LPHN1, or ADGRL1) [35], the adhesion GPCR type L1 that binds several protein ligands, including α-latrotoxin (αLTX) [36,37], teneurin-2 [38], and FLRT3 [39]. This lack of endogenous LPHN1 permits transgenic expression of its mutants designed to investigate intracellular signaling associated with this receptor. On the other hand, these cells possess different GPCR-coupled pathways [27,40,41] and demonstrate robust responsiveness to GPCR ligands [42,43]. Combined with exceptional transfection efficiency, these features of NB cells facilitate precise manipulation of Ca^2+^ regulatory components and real-time monitoring of Ca^2+^ dynamics using genetically encoded indicators [27]. While lacking the full synaptic complexity of primary neurons, NB cells offer an optimal balance between physiological relevance and experimental practicality for mechanistic studies of receptor-mediated Ca^2+^ signaling [44].

In this study, we exploit these advantages to investigate how LPHN1 couples extracellular stimuli to SOCE activation [45]. Using differentiated NB cells expressing LPHN1 or its non-signaling mutant, we catalog the changes in the expression of a range of proteins involved in Ca^2+^ homeostasis and SOCE. By stimulating the cells with the αLTX mutant LTX^N4C^ [46], we demonstrate receptor-mediated release of stored Ca^2+^ and subsequent SOCE engagement. Our results not only elucidate the interplay between GPCR-induced SOCE and CICR in shaping presynaptic Ca^2+^ transients but also establish a framework for understanding how extracellular signals integrate with presynaptic Ca^2+^ homeostasis under physiological and pathological conditions.

## 2. Results

### 2.1. Differentiation Changes Cell Morphology and Receptor Expression

**Figure 1:**
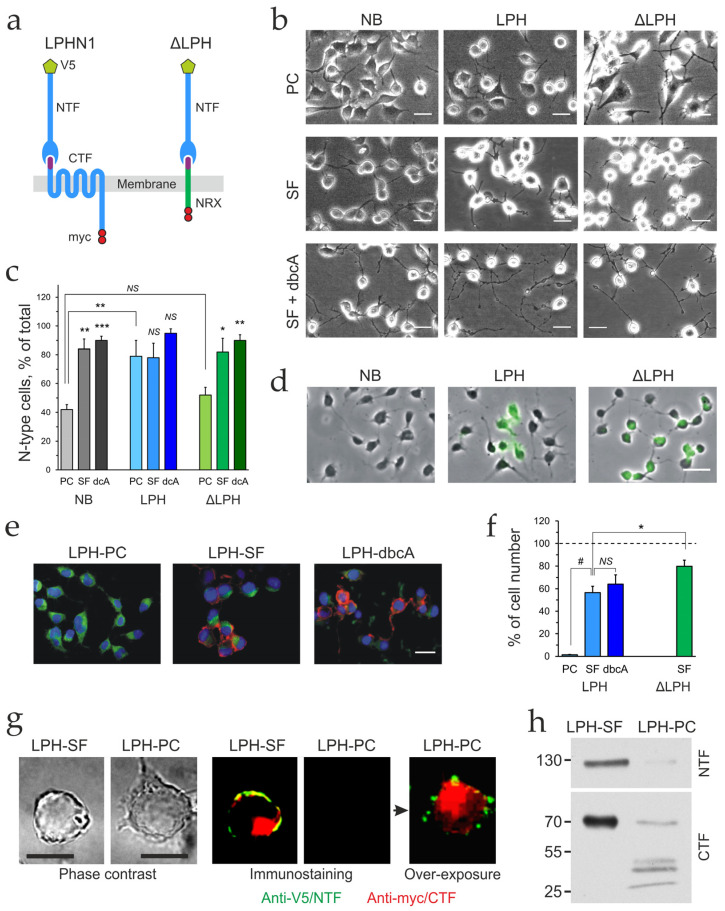
Changes in cell morphology and LPHN1 expression with cell differentiation. (**a**) Wild-type and mutant receptor constructs that were used to stably transfect NB cells. LPHN1 is self-cleaved into two fragments (NTF and CTF). The mutant construct ΔLPH, consisting of the NTF and a C-terminal segment of neurexin I (NRX), is also self-cleaved. The NTF and CTF contain immunological tags (V5 and myc) for reliable detection. (**b**) A shift to neuron-like morphology induced in NB, LPH, and ΔLPH cells by construct expression and differentiation. The cells were maintained in complete medium or differentiated by 48 h incubation in SF medium ± 1 mM dbcAMP (dbcA). Neuron-like (N-type) cells have compact somata and show high phase contrast (white halo). The scale bars are 30 μm. (**c**) Per cent of N-type cells in each culture upon differentiation under respective conditions. (**d**) Expression of the receptor constructs in stably transfected LPH and ΔLPH cells. NB, LPH, and ΔLPH cells were differentiated by serum deprivation for 24 h, fixed, and labeled with an anti-V5 antibody and Alexa Fluor-488-conjugated IgG (green). The scale bar is 30 μm. (**e**) LPH cells overexpress LPHN1 upon differentiation. LPH cells were maintained in complete medium or differentiated in SF medium ± dbcAMP, then fixed, permeabilized, and labeled with anti-myc (red) and anti-NFH (green) antibodies, and a nuclear stain, DAPI (blue). The scale bar is 30 μm. (**f**) Per cent of LPH and ΔLPH cells (PC, proliferating; SF, differentiated by serum deprivation; dbcA, differentiated by dbcAMP) overexpressing respective constructs. (**g**) Low-level LPHN1 expression in proliferating LPH cells. LPH-SF and LPH-PC cells were pretreated as in (**c**) and immunostained with fluorescently labeled anti-V5 (green) and anti-myc (red) antibodies. Left, phase contrast images of individual cells from respective cultures. Middle, immunostaining of the same cells with regular image exposure/enhancement. Right, over-exposure and enhancement of the middle image demonstrates that both the NTF and CTF are present in proliferating LPH cells. The scale bar is 10 μm. (**h**) LPHN1 degradation in proliferating LPH cells. Approximately 1 × 10^5^ LPH-SF cells and 4 × 10^5^ LPH-PC cells were analyzed by SDS-electrophoresis and Western blotting using antibodies against the NTF and CTF. Relative molecular masses in kDa are shown on the left. The graphs in (**c**,**f**), show the means ± SE; the number of experiments was *n* = 6 with 8–37 fields of view (*N* = 48–95; total number of cells per culture 213–669) (**c**) and *n* = 3 with 3–9 fields of view imaged (*N* = 9–15) (**f**). Asterisks indicate statistical significance of differences (one-way ANOVA) between the respective differentiated and proliferating cells or as indicated by lines; *, *p* < 0.05; **, *p* < 0.01; ***, *p* < 0.001; #, *p* < 0.0001; NS, non-significant.

For dissecting the role of intracellular signaling in LTX^N4C^-induced response, two receptor constructs were used (Figure 1a): the full-length LPHN1 and its chimeric mutant ΔLPH. In ΔLPH, the native CTF was replaced with the transmembrane domain and C-terminus of neurexin I. As a result, ΔLPH retains the ability to bind αLTX and LTX^N4C^ with its NTF but loses the ability to signal via G proteins. N- and C-terminal immunological tags (V5 and myc) were added to both constructs for reliable detection (Figure 1a). These constructs were used to transfect NB cells, which were then extensively selected and cell-sorted to produce two stable cell lines expressing LPHN1 (LPH cells) or ΔLPH (ΔLPH cells).

In our experiments under high-serum conditions, undifferentiated NB cells rapidly proliferated and exhibited an adherent, epithelial-like morphology (Figure 1), as also reported previously [27]. The proliferating culture contained two distinct types of cells, with neuronal and amoeboid stem cell features (Figure 1b, NB-PC), which were similar to the N-type and S-type cells found in human neuroblastoma lines [47]. N-type neuroblastic cells had small, round cell bodies with a small amount of cytoplasm and several processes (neurites). Substrate-adherent S-type cells possessed broad and flattened cell bodies with shorter processes, and exhibited non-neuronal properties similar to glial cells, Schwann cells, and melanocytes.

A key feature of NB cells is their ability to differentiate upon serum reduction and cAMP elevation and develop strong neuronal characteristics [27,42,48,49]. To elucidate how the expression of the receptor constructs and cell differentiation affected the cells, we quantified the number of N-type cells and the number and length of neurites. The three cell lines were treated with a serum-free (SF) medium alone or with the addition of dibutyryl cyclicAMP (dbcAMP) (Figure 1b). Proliferating NB cells (NB-PC) and ΔLPH cells (ΔLPH-PC) contained a roughly equal mixture of S- and N-type cells, whereas proliferating LPH cells (LPH-PC) were predominantly of the N-type (80% in LPH-PC vs. 41% in NB-PC, *p* < 0.007; vs. 52% in ΔLPH-PC, *p* = 0.037) (Figure 1b, top row; 1c). Serum-deprivation significantly increased the proportion of N-type cells in SF-differentiated cultures (83% in NB-SF vs. 41% in NB-PC, *p* < 0.0001, and 84% in ΔLPH-SF vs. 52% in ΔLPH-PC, *p* < 0.009) but did not further increase the number of N-type cells in SF-differentiated LPH cultures (79% in LPH-SF vs. 80% in LPH-PC) (Figure 1b, middle row; 1c). The addition of dbcAMP to SF medium increased the population of N-type cells insignificantly in NB and ΔLPH cells only (91% in NB-dbcA vs. 41% in NB-PC, *p* < 0.0001; 96% LPH-dbcA vs. 80% in LPH-PC, *p* = 0.15; 84% in ΔLPH-dbcA vs. 52% in ΔLPH-PC, *p* 0.002) (Figure 1b, bottom; 1c).

In PC and SF cultures, N-type cells displayed fewer neurites than S-type cells (2.9 ± 0.1 vs. 4.2 ± 0.2 neurites per cell, respectively; *p* = 0.0007) (Figure 1b, top, middle rows; Figure S1a), but they were generally longer (25 ± 2.3 vs. 19 ± 0.8 μm, respectively; *p* = 0.039) (Figure S1b). Receptor expression or cell differentiation had no effect on the average number of neurites per cell (Figure S1a). There was no change in neurite length in S-type cells, suggesting that they were unable to undergo differentiation under any treatment. Neurite length in N-type cells also was not affected by SF-differentiation, but serum deprivation with dbcAMP dramatically increased neurite length only in N-type NB and LPH cells (Figure 1b, bottom row; Figure S1b). The percentage of N-type NB-dbcA and LPH-dbcA that expressed neurites over 50 μm, an indicator of neuronal differentiation, was 36 ± 8.5 and 42 ± 2.1%, respectively (NB-dbcA and LPH-dbcA vs. all other cells: *p* > 0.001) (Figure S1c).

In summary, LPH cells displayed morphological features that were indicative of a more neuronal phenotype, compared to NB and ΔLPH cells. The higher number of N-type cells in undifferentiated LPH cells and the increased length of neurites upon SF/dbcAMP treatment suggested that LPHN1 expression supported neuronal differentiation of NB cells.

We next evaluated receptor expression in LPH and ΔLPH cells. While immunostaining clearly demonstrated the presence of the target proteins in both transgenic lines (but not in the wild-type NB cells), only a proportion of transfected cells produced a sufficient amount of receptor for immunostaining (Figure 1d). Furthermore, proliferating LPH cells did not appear to express the receptor until they were differentiated using SF medium (Figure 1e). Even after differentiation with or without dbcAMP, only ~54% of LPH cells showed LPHN1 immunostaining (Figure 1f). By contrast, ~80% of ΔLPH cells differentiated in SF medium demonstrated detectable ΔLPH staining (Figure 1f).

These observations implied that the LPHN1 transgene was repressed in most proliferating and in some differentiated LPH cells. However, this was inconsistent with the constitutive character of the strong cytomegalovirus promoter driving LPHN1 expression and the role of the receptor in morphological changes in proliferating LPH cells, as described above. We therefore hypothesized that LPHN1 was expressed but rapidly degraded in dividing cells. To test this, the images of proliferating LPH cells that appeared to lack LPHN1 immunostaining were enhanced, revealing the presence of small amounts of receptor (Figure 1e). Importantly, only the NTF remained on the cell surface, while the CTF was primarily localized to intracellular organelles, likely lysosomes (Figure 1e, bottom), indicating that it was recycled separately from the NTF, as previously reported [50].

This hypothesis was tested by immunocytochemical analysis of the subcellular distribution of receptor fragments in LPH-SF and LPH-PC cells (Figure S1d). In differentiated LPH-SF cells, 96.3 ± 0.9% of the NTF and 36.3 ± 4.3% of the CTF localized to the plasma membrane. These proportions were dramatically reduced in proliferating LPH cells. In addition to an overall low level of LPH expression, only 19.7 ± 1.9% of the NTF and 7.6 ± 1.5% of the CTF were detected in the plasma membrane. Furthermore, 59.29 ± 7.18% of the intracellular CTF was localized to lysosomes, identified by LysoTracker staining (Figure S1e), while the remaining 40.71 ± 7.18% was present in non-lysosomal vesicular compartments. Notably, 86.5 ± 4.1% of lysosomes contained the CTF, suggesting that receptor was continuously expressed in LPH-PC cells. A substantial overlap between lysosomal and CTF staining was confirmed by quantitative colocalization analysis, which yielded Manders’ split coefficients M1 and M2 of 0.8 and 0.39, respectively. Finally, the specificity of the immunostaining was verified by Western blotting (Figure 1h), which demonstrated not only smaller amounts of receptor fragments in LPH-PC cells than in LPH-SF cells but also substantial CTF degradation in the proliferating cells.

We conclude that proliferating LPH cells express LPHN1 but actively recycle it to prevent background signaling and a consequent N-type differentiation. Serum deprivation, with or without dbcAMP, causes the cells to cease division, a phase of the cell cycle when protein ubiquitination and proteasome-mediated protein degradation are most active [51,52,53,54]. We propose that when the cells enter the growth stage, this allows LPHN1 to escape rapid degradation and accumulate.

Based on these results, all subsequent experiments investigating LPHN1’s role in Ca^2+^_cyt_ dynamics used SF medium to differentiate receptor-expressing cells, but omitted dbcAMP due to its relatively minor additional effect on receptor expression.

### 2.2. LPHN1 Activation by LTX^N4C^ Elevates Ca^2+^_cyt_ in the Presence of Ca^2+^_e_

The αLTX mutant LTX^N4C^ was designed in the laboratory of Thomas C. Südhof [46] and subsequently extensively used to characterize LPHN1-mediated signaling in neurons, endocrine cells, and transfected NB cells [35,55,56,57,58,59,60,61]. In contrast to αLTX, LTX^N4C^ lacks the ability to form cation-permeable pores in the cell membrane [62] and permits LPHN1 stimulation in the absence of the non-specific effects of αLTX pores.

To specifically identify which actions of the mutant toxin involved intracellular signaling from the receptor, we monitored Ca^2+^_cyt_ changes induced by LTX^N4C^ in differentiated LPH and ΔLPH cells, following a protocol typically employed to measure Ca^2+^ release and SOCE induced by thapsigargin (TG) (Figure S2a). According to this protocol, cells are loaded with the Ca^2+^-sensing dye Fluo-4, and their fluorescence is continuously recorded. Stimulating the cells with TG in a Ca^2+^_e_-free medium reveals Ca^2+^ release from intracellular stores, while the subsequent addition of 2 mM Ca^2+^_e_ shows SOCE manifested as a large transient peak of [Ca^2+^]_cyt_ (Figure S2a, left). At the end of SOCE, a new post-SOCE Ca^2+^_cyt_ equilibrium (PostEq) is established, which in the case of TG often equals the basal [Ca^2+^]_cyt_ in the presence of Ca^2+^_e_. As reported previously [45], αLTX also causes Ca^2+^ release and SOCE (Figure S2a, middle), which are mediated by LPHN1, although this action is complicated by αLTX forming a membrane pore.

**Figure 2:**
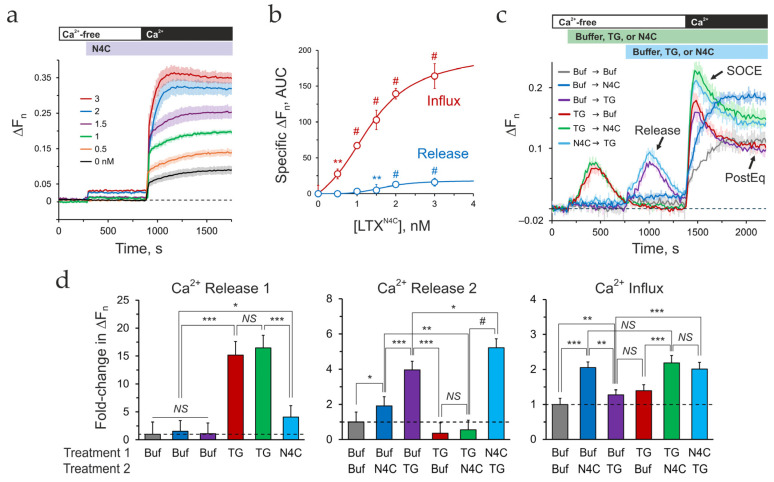
LTX^N4C^ induces an increase in [Ca^2+^]_cyt_ via LPHN1 but does not involve the TG-sensitive Ca^2+^ stores. LPH-SF cells were loaded with the Ca^2+^-sensing dye Fluo-4 AM and recorded in a multi-well fluorescent plate reader, and stimulated as shown above the traces (**a**,**c**). The dye-loading protocol, applicable to all experiments with Fluo-4, is described in detail in Section 4.7.1. Initial incubation in a nominally Ca^2+^-free medium was followed by the addition of LTX^N4C^ or TG, and subsequent addition of 2 mM Ca^2+^. (**a**) Dose dependence of changes in Ca^2+^_cyt_ fluorescence induced by 0–3 nM LTX^N4C^. (**b**) Quantification of the data in (**a**) after subtracting the basal Ca^2+^ fluorescence. LTX^N4C^ causes a small increase in Ca^2+^_cyt_ in the Ca^2+^-free buffer and a large Ca^2+^_e_ influx after Ca^2+^ addition. Both effects are LTX^N4C^ dose-dependent. (**c**) LTX^N4C^ and TG induce Ca^2+^ release and influx by acting through different mechanisms. Normalized Ca^2+^ fluorescence changes triggered by 1 nM LTX^N4C^ before or after treatment with 0.3 μM TG in the absence and then presence of Ca^2+^_e_. Buf, buffer (no stimulus). The main phases of TG action are also indicated by arrows: Ca^2+^ release, SOCE peak, and post-SOCE Ca^2+^_cyt_ equilibrium (PostEq). (**d**) Specific (above basal) fold-changes in Ca^2+^ fluorescence induced by LTX^N4C^ or TG during Treatments 1 and 2 in the Ca^2+^-free medium (Release 1 and Release 2), and after re-addition of Ca^2+^ (Influx). For illustration, Δ*F*_n_ values were aggregated over time for each phase as AUCs and normalized to Δ*F*_n_ in unstimulated cells (Buf → Buf). The dashed lines show the basal level (fold-change 1). (**a**–**d**) The data are the means of *n* = 3–5 experiments ± SE (**a**,**b**) or SD (**d**,**c**). Asterisks show statistical significance of the differences between indicated conditions, tested using FANOVA (**b**) or one-way ANOVA (**d**); *, *p* < 0.05; **, *p* < 0.01; ***, *p* < 0.001; #, *p* < 0.0001; NS, non-significant.

Stimulation of LPH-SF cells by LTX^N4C^ in the absence of Ca^2+^_e_ led to a fast but small increase in [Ca^2+^]_cyt_, which occurred within 15 s of toxin addition and was sustained at the same level during Ca^2+^-free incubation (Figure 2a and Figure S2a, right). This rise in Ca^2+^_cyt_ represented LTX^N4C^-induced Ca^2+^ Release. This LTX^N4C^ effect was dose-dependent (Figure 2b).

Subsequent addition of 2 mM Ca^2+^ to the medium induced a fast surge in [Ca^2+^]_cyt_, followed by a gradual increase in Ca^2+^_cyt_ (Figure 2a). While unstimulated LPH cells reacted to Ca^2+^ addition similarly, response to LTX^N4C^ was much more robust and dose-dependent (Figure 2b). The slow phase of this increase in Ca^2+^_cyt_ developed over ~120–200 s, did not decay within the time of the experiment, and displayed a small transient peak at high LTX^N4C^ concentrations (Figure 2a), which was reminiscent of the SOCE induced by TG or αLTX (Figure S2a). However, due to the lack of a transient peak of Ca^2+^ fluorescence, quantification of SOCE separately from the PostEq was not possible, and the overall effect was determined as LTX^N4C^-induced Ca^2+^ influx above basal (Figure 2b and Figure S2a).

The specificity of LTX^N4C^ action was demonstrated by applying LTX^N4C^ to proliferating LPH cells and differentiated ΔLPH-SF cells (Figure S2b,c). Consistent with the low level of surface-exposed LPHN1 in proliferating cells (Figure 1g and Figure S1d,e), LPH-PC cells reacted to the toxin by both releasing some Ca^2+^ and allowing a small Ca^2+^ influx during respective stages of the protocol. Functional data analysis (FDA) [63] revealed significant difference between the control and LTX^N4C^ stimulation in proliferating LPH-PC cells (*p* = 0.038, FANOVA) (Figure S2b). Pointwise tests confirmed differences during both the Ca^2+^ release phase (300–1200 s) and Ca^2+^ influx phase (1250–2000 s). As expected, the reaction of differentiated ΔLPH cells (ΔLPH-SF) to LTX^N4C^ did not differ from that of control (unstimulated) ΔLPH-SF cells at both protocol stages (Figure S2c).

The Ca^2+^ release and influx stimulated by LTX^N4C^ via LPHN1 exhibited two unexpected characteristics.

First, the Ca^2+^ level during the release phase was both low and constant, implying a weak signal or a limited Ca^2+^ store. To determine whether LTX^N4C^ acts on ER Ca^2+^ stores, we stimulated LPH-SF cells with LTX^N4C^ and TG, a sarcoplasmic/endoplasmic reticulum Ca^2+^-ATPase (SERCA) inhibitor that depletes ER stores. The two stimulants were applied sequentially during the Ca^2+^-free phase, followed by Ca^2+^_e_ addition (Figure 2c). As anticipated, TG consistently produced classical Ca^2+^ dynamics: Ca^2+^ release, SOCE, and PostEq (Figure 2c; compare Buf → TG with TG → Buf). Unexpectedly, when LTX^N4C^ was applied first, it did not diminish TG-induced Ca^2+^ release or SOCE but instead had an additive effect with TG (Figure 2c,d; compare Buf → TG with N4C → TG). Even more strikingly, pre-treatment with TG significantly inhibited the subsequent LTX^N4C^-induced Ca^2+^ release but still resulted in a Ca^2+^ influx substantially stronger than that induced by TG alone (Figure 2c,d; compare Buf → N4C with TG → N4C and TG → Buf).

To explain the Release data, we hypothesized that LTX^N4C^ signaling via LPHN1 mobilizes a TG-insensitive Ca^2+^ store that can exchange Ca^2+^ with the ER. When LTX^N4C^ is applied before TG, the ER remains full, leading to additive Ca^2+^ release from both stores. However, when TG is applied first, it depletes the ER, which subsequently draws Ca^2+^ from the small LTX^N4C^-sensitive store. This reduces the amount of Ca^2+^ available for subsequent release by LTX^N4C^. Consistent with this model, the SOCE phase data indicate that the TG- and LTX^N4C^-sensitive stores activate Ca^2+^ influx independently and so have an additive effect.

Second, the LTX^N4C^-induced Ca^2+^ influx lacked the large transient peak typically associated with the synchronized opening and inactivation of SOCCs, as seen with TG or αLTX treatment, prompting us to investigate its underlying cause. While the distinct Ca^2+^ stores mobilized by LTX^N4C^ and TG likely contribute to the differing influx patterns (see Section 3), a technical factor may also be responsible. Ca^2+^ fluorescence traces recorded in a microplate fluorometer represent integrated population-level signals from 500 to 1000 cells in each well. Although individual cells may exhibit transient SOCE peaks, when these responses are desynchronized across the population, they will average out to a smooth, gradual increase in bulk measurements—a known limitation of such assays [64]. To overcome this constraint and resolve Ca^2+^ dynamics at the single-cell level, we turned to confocal fluorescence microscopy.

### 2.3. Ca^2+^ Signaling Induced by LPHN1 Activation in Individual Cells

**Figure 3:**
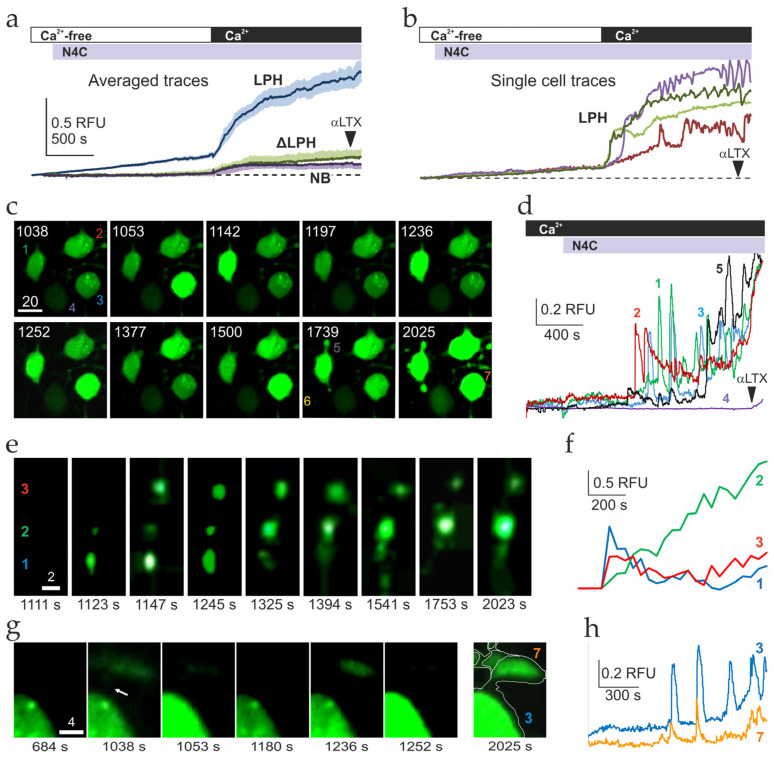
Changes in Ca^2+^_cyt_ levels in individual NB, LPH, and ΔLPH cells upon stimulation with LTX^N4C^. The cells were loaded with the Ca^2+^ sensing dye Fluo-4 AM, recorded under a confocal microscope, and stimulated as indicated by bars at the top. (**a**,**b**) The cells were incubated in a Ca^2+^-free medium, when the background fluorescence *F*_0_ was recorded. The cells were then stimulated with 2 nM LTX^N4C^, supplied with 2 mM Ca^2+^_e_, and finally permeabilized with 2 nM αLTX to record the maximal Ca^2+^ fluorescence *F*_max_ (arrowheads). All data were normalized to *F*_0_ and *F*_max_, as described in Section 4.7.2. (**a**) Average traces ± SE from 22 to 26 individual NB, LPH and ΔLPH cells. RFU, normalized relative fluorescence units. (**b**) Individual fluorescence traces ± SE from randomly selected LPH cells. The data are representative of *n* = 4 independent experiments with 20–30 individual cells recorded in each (*N* = 80–120). (**c**–**h**) The LPH cells were incubated in the presence of 2 mM Ca^2+^_e_, stimulated with 2.5 nM LTX^N4C^, and permeabilized with 2.5 nM αLTX (arrowhead). (**c**) Selected time-lapse fluorescent images of a group of cells (1–4). The numbers indicate the time in seconds from the beginning of recording; the scale bar is 20 μm. (**d**) Fluorescence intensity profiles of individual cells and varicosities from (**c**). LTX^N4C^, Ca^2+^, and αLTX additions are shown by arrowheads. The colored numbers indicate corresponding cells or varicosities in (**c**). Note that the undifferentiated cell 4 did not respond to either toxin. (**e**) Selected fluorescent images of neurite 6 from (**c**) containing varicosities (1–3), which independently respond to LTX^N4C^ stimulation. The scale bar is 2 μm. (**f**) Relative fluorescence within the individual varicosities from (**e**). (**g**) Selected fluorescent images of a varicosity (V) physically and functionally linked to cell 3 from (**c**). The scale bar is 4 μm; the arrow in image 2 in panel (**g**) indicates a Ca^2+^ transient in a neurite connecting the varicosity and cell; the last image in series (**g**) shows the same neurite traced after permeabilization with αLTX. (**h**) Respective relative fluorescence intensity profiles. Note that the Ca^2+^ signal in the varicosity precedes the signal within the cell. The experiment is a representative of *n* = 9 independent experiments, with 4–25 individual cells recorded (*N* = 36–160), which showed similar results.

Differentiated LPH, ΔLPH, and NB cells were loaded with Fluo-4 AM and treated according to the standard protocol, while confocal images of multiple cells were frequently acquired under a confocal fluorescent microscope. By measuring the brightness of all cells in a series of time-lapse images, average Ca^2+^ fluorescence traces were produced, which appeared very similar to the bulk fluorescence traces described above (Figure 3a). The average traces show that LPH cells reacted to LTX^N4C^ by releasing Ca^2+^ during the Ca^2+^_e_-free phase, and by gradually increasing [Ca^2+^]_cyt_ after Ca^2+^_e_ addition. By contrast, ΔLPH and NB average traces did not demonstrate Ca^2+^ release but only showed a low basal Ca^2+^_cyt_ influx after Ca^2+^_e_ addition (Figure 3a).

However, Ca^2+^ fluorescence traces of individual cells obtained from the same confocal images revealed a more complex behavior (Figure 3b). During the Ca^2+^ influx phase, single LPH cells showed sharp Ca^2+^_cyt_ peaks, combined in some cells with [Ca^2+^]_cyt_ oscillations. A key feature of these Ca^2+^ spikes and oscillations was the lack of their synchronization between individual cells (Figure 3b). Individual ΔLPH and NB cells showed no Ca^2+^ oscillations (Figure S3a).

To extract more detailed information from single-cell recordings, we carried out confocal fluorescent imaging of LPH-SF and ΔLPH-SF cells at a higher magnification and added Ca^2+^_e_ before LTX^N4C^ to prevent any Ca^2+^ signal synchronization (Figure 3c and Figure S3b). Ca^2+^ fluorescence traces (Figure 3d) demonstrate that, after a delay of 10.7 ± 0.6 min, LTX^N4C^ induced high-amplitude Ca^2+^_cyt_ spikes. Using Equation (3) (Section 4.7.2), peak [Ca^2+^] in these spikes was estimated to average 2.2 ± 1.1 μM but reached 27 μM Ca^2+^_cyt_ in some cells. In many cells, these spikes occurred with an imperfect periodicity of 113.8 ± 18.1 s, while other cells demonstrated Ca^2+^_cyt_ oscillations with a lower frequency (average period 73.9 ± 6.4 s) and amplitude (average peak Ca^2+^ concentration 127.6 ± 6.7 nM). These effects were specific to LPH cells, where they occurred in 61 ± 5% of cells, and were never observed in ΔLPH cells (Figure S3b,c). In addition, 25 ± 3.2% of LPH-SF cells did not undergo full differentiation and, therefore, only expressed low amounts of receptors. Such cells showed no LTX^N4C^-induced Ca^2+^ influx but reacted to permeabilization with αLTX by a very small increase in Ca^2+^_cyt_ (Figure 3c, cell 4). Thus, LTX^N4C^ produced in LPH cells a desynchronized SOCE, which was likely combined with CICR.

The single-cell Ca^2+^ fluorescence recordings demonstrated interesting features of LTX^N4C^ effect. For example, individual varicosities, which were located on the same neurite within 3–4 μm from each other (Figure 3e), showed different and independent patterns of Ca^2+^ regulation (Figure 3f). Other varicosities were apparently functionally connected to the cell body (Figure 3g), so that periodic small Ca^2+^ spikes occurring within the varicosity were transmitted to the cell body, where they were amplified, producing, after a short delay, large Ca^2+^_cyt_ peaks of higher amplitudes (Figure 3h). This suggests that varicosities, which in differentiated NB cells resemble immature nerve terminals [65], contain both LPHN1 and its downstream signaling machinery, and can be activated by LTX^N4C^ independently of each other or the cell body.

A key characteristic of LPHN1-mediated LTX^N4C^ action was its dependence on Ca^2+^_e_ and delayed onset. When LTX^N4C^ was applied in the presence of Ca^2+^_e_, calcium spikes appeared after a 15–20 min delay (Figure 3d). However, if cells were first preincubated with the toxin, subsequent Ca^2+^_e_ addition triggered an immediate strong Ca^2+^ influx, which later led to Ca^2+^ spikes (Figure 3b). Similarly, the rate of the gradual overall increase in [Ca^2+^]_cyt_ (another feature observed after the onset of toxin’s action) (Figure 3a,b,d,f,h) also depended on the order of reagent addition: it was slow when LTX^N4C^ followed Ca^2+^_e_ (Figure 3d,h), but rapid when LTX^N4C^ preceded it (Figure 3a,b). Together, these findings indicate that LTX^N4C^ binding to LPHN1 primes the opening of plasma membrane Ca^2+^ channels and activates a concerted mechanism of SOCE and CICR (see Section 3).

Based on these results, we designed subsequent experiments to identify the specific Ca^2+^ channels and Ca^2+^ sensor proteins involved in SOCE that are uniquely expressed in differentiated LPH cells.

### 2.4. Expression of SOCE-Associated Proteins

**Figure 4:**
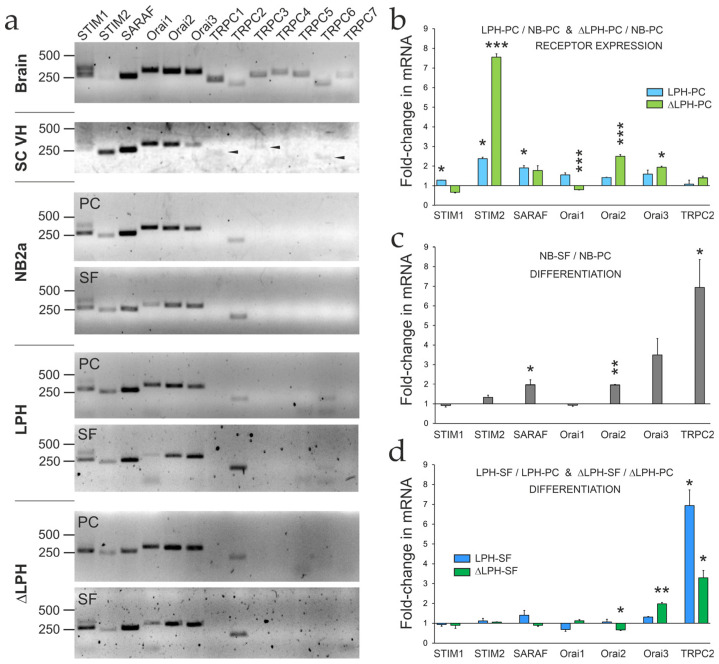
Expression of SOCE-associated proteins in proliferating and differentiated NB, LPH, and ΔLPH cells. (**a**) Agarose gel analysis of RT-PCR products obtained on cDNA prepared from mouse forebrain, the ventral horn of the lumbar segment of mouse spinal cord (SC VH), NB cells (NB), and two receptor-expressing cell lines (LPH and ΔLPH), which were proliferating (PC) in complete medium or differentiated for 48 h in SF medium (SF). The following SOCE-associated mRNAs were detected (expected band size, bp): STIM1 (296), STIM2 (256), SARAF (280), Orai1 (335), Orai2 (327), Orai3 (318), and TRPC2 (181). TRPC6 (179) was detected in LPH cells only. The arrowheads show the low levels of TRPC1, 3 and 6 in the spinal cord. An additional band found in all reactions targeting STIM1 cDNA did not affect the relative quantification of STIM1. PC: proliferating cells; SF: cells differentiated in SF medium. Numbers on the left show the sizes of selected markers in bp. The gels are representative of *n* = 3 experiments with 3 replicates (*N* = 9), which gave similar results. (**b**–**d**) Relative quantification of SOCE proteins mRNA levels based on the results of qRT-PCR. (**b**) Fold-changes in mRNA levels induced by LPH or ΔLPH expression in proliferating cells, relative to NB cells. (**c**) Fold-changes in mRNA levels induced by SF-differentiation of NB cells, relative to proliferating NB cells. (**d**) Fold-changes in mRNA levels induced by SF-differentiation of LPH and ΔLPH cells, relative to respective proliferating cells. In (**b**–**d**), the bars show the means ± SE from *n* = 3–4 independent experiments performed in triplicates (*N* = 9–12). Asterisks indicate statistical significance of differences (tested by one-way ANOVA) between proliferating LPH or ΔLPH cells and NB cells in (**b**), differentiated and proliferating NB cells in (**c**), and differentiated LPH or ΔLPH cells and respective proliferating cells in (**d**). Non-significant differences in (**c**,**d**) are not shown for simplicity; *, *p* < 0.05; **, *p* < 0.01; ***, *p* < 0.001.

To define the repertoire of SOCE-associated proteins in proliferating and differentiated NB, LPH, and ΔLPH cells, we analyzed key sensor and channel proteins (Figure 4). These included the ER-resident Ca^2+^ sensors STIM1, STIM2, and the regulatory factor SARAF, which fine-tunes SOCE and prevents excessive Ca^2+^ influx [66,67]. We also assessed potential SOCCs: Orai1–3 and TRPC1–7 channels [68]. While Orai1–3 are primary contributors to SOCE, some TRPC channels (particularly TRPC1/4/5) can also play a role [69,70].

Endogenous mRNAs encoding SOCE proteins were first identified by standard RT-PCR and agarose gel electrophoresis (Figure 4a), which confirmed the specificity of the primers used. All the cell lines tested expressed the ER-membrane proteins (STIM1–2 and SARAF) and some plasma membrane channels (Orai1–3 and TRPC2), while only LPH cells contained TRPC6. Expression profiles in all cells and especially LPH-PC were similar to those in motor neurons from the ventral horn of a mouse spinal cord (Figure 4a), except that the neurons lacked TRPC2 but expressed low levels of TRPC3 (Figure 4a). This supported the relevance of LPH cells as a model system for studying the molecular components of Ca^2+^ regulation by LPHN1, with the possibility of extending certain conclusions to neurons.

To determine the effect of receptor expression and cell differentiation on the levels of SOCE-associated proteins, qRT-PCR was performed on proliferating and differentiated NB, LPH, and ΔLPH cells (Figure S4). Overall, all cells contained approximately 10-fold more SARAF than STIM and Orai proteins, while expressing very low amounts of TRPC2.

Receptor expression upregulated most mRNAs, but to varying extents (Figure 4b). STIM1 and Orai1 reached higher levels in LPH cells than in ΔLPH cells. SARAF and Orai3 were equally upregulated in both receptor-expressing cells, while STIM2, Orai2, and TRPC2 were especially increased in ΔLPH cells.

Differentiation of cells by serum deprivation (Figure 4c,d) caused an upregulation of SARAF, Orai2, Orai3, and TRPC2 in NB-SF cells, but only Orai3 and TRPC2 in receptor-expressing cells. STIM1, STIM2, Orai2, and Orai3 were less affected by differentiation in receptor-expressing cells than NB cells, possibly because they were already significantly upregulated in proliferating LPH or ΔLPH cells. Differentiation of LPH cells did not significantly affect individual SOCE mRNAs, except for TRPC2, but it was also strongly upregulated in ΔLPH-SF and NB-SF cells.

In summary, STIM2 and Orai2 exhibited the strongest specific upregulation in response to receptor expression. Importantly, Orai2 is much more strongly expressed in the brain than in other tissues [71], suggesting that it is likely to be associated with LPHN1 signaling. The role of these proteins in LPHN1-mediated LTX^N4C^ action was further investigated by RNA interference (RNAi).

### 2.5. The Role of Orai2 in LPHN1-Mediated LTX^N4C^ Action

shRNA-mediated knockdown of Orai2 mRNA was performed by transfecting LPH-SF cells with plasmids encoding small hairpin RNAs (shRNAs). Four previously uncharacterized Orai2-targeting shRNA constructs (sh1–4) were evaluated. The level of Orai2 mRNA in shRNA-transfected cells was assessed by qRT-PCR and compared to that in untransfected cells (Figure S5a). Plasmids encoding sh1 and sh2 produced the highest level of Orai2 mRNA degradation. Based on the positive knockdown results, sh1 and sh2 were compared in relation to their effect on [Ca^2+^]_cyt_ regulation and the expression of other SOCE-associated genes.

Bulk Ca^2+^ fluorescence recordings were performed on LPH-SF cells transfected with sh1 or sh2, loaded with Fluo-4 AM, and stimulated with TG using the standard stimulation protocol (Figure S5b). The sh1 plasmid caused a significant decrease in TG-induced Ca^2+^ release and also attenuated SOCE and PostEq (Figure S5c), while the sh2 plasmid had no effect on TG actions.

**Figure 5:**
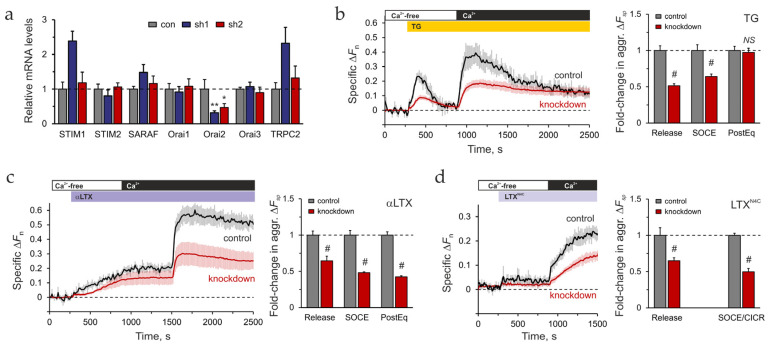
Knockdown of Orai2 mRNA decreases Ca^2+^_cyt_ levels during the Ca^2+^ release and influx phases. (**a**) The effect of sh1 or sh2 shRNA expression on the mRNA levels of SOCE-associated proteins. LPH cells were transfected with the sh1 or sh2 plasmid, allowed to grow for 24 h, and then differentiated in SF medium for 48 h. The mRNA levels were determined using qRT-PCR and normalized to β-actin and untransfected (control) values for each mRNA. Non-significant differences are not shown for simplicity. (**b**–**d**) The effect of Orai2 knockdown on stimulated changes in Ca^2+^_cyt_. To restrict Ca^2+^ fluorescence detection to knockdown cells, LPH cells were co-transfected with the sh2 and GCaMP6S plasmids. Subsequently, cells were grown for 24 h, differentiated in SF medium for 48 h, and stimulated (as shown by the bars above) with 0.3 μM TG (**b**), 1 nM αLTX (**c**), or 2 nM LTX^N4C^ (**d**). Finally, the cells were incubated in 2 mM Ca^2+^_e_. (**Left panels**): Averaged traces of specific changes in Ca^2+^_cyt_ fluorescence after the subtraction of basal fluorescence and normalization to baseline (Figure S5e). The dashed lines indicate 0. (**Right panels**): Fold-changes in aggregated specific Ca^2+^ fluorescence (area under the curve, AUC, above background) in Orai2-knockdown cells relative to control (untransfected) cells during the Release, SOCE, and PostEq phases. For illustration, Δ*F*_n_ values were aggregated over time for each phase as AUCs. The dashed lines indicate the control level (fold-change 1). The data are the means of *n* = 3 experiments ± SD (**a**) or SE (**b**–**d**). Statistical tests applied were one-way ANOVA (**a**) and FANOVA (**b**–**d**). Asterisks denote statistical significance of the difference between knockdown and control cells for each condition; *, *p* < 0.05; **, *p* < 0.01; #, *p* < 0.0001; NS, non-significant.

Surprisingly, while sh1 and sh2 inhibited Orai2 expression to a similar extent, only sh1 significantly upregulated other SOCE mRNAs (STIM1, SARAF, and TRPC2) (Figure 5a). This could be due to off-target effects of sh1, which would hinder the interpretation of any results. Consequently, sh2 was selected for Orai2 knockdown studies.

It is important to note that mRNA quantification likely underestimates the extent of knockdown in individual cells. Since the mRNA analysis was performed on the whole culture, while only 30–40% of cells were transfected with the sh plasmids, the data in Figure S5a and Figure 5a likely reflect the complete degradation of Orai2 mRNA in the successfully transfected cells. To overcome this discrepancy, we used a genetically encoded Ca^2+^ indicator, GCaMP protein. When co-transfected with the sh plasmid, both Orai2 mRNA interference and GCaMP expression occur in the same cells, allowing Ca^2+^ fluorescence recordings to be limited to knockdown cells.

First, to compare the performance of GCaMP and Fluo-4 under our experimental conditions, we analyzed their sensitivity and response kinetics to cytosolic Ca^2+^ changes. Differentiated NB cells were either transfected with GCaMP6S or loaded with Fluo-4 AM, then stimulated with TG. As shown by the baseline-normalized traces (Figure S5d), GCaMP exhibited a lower response amplitude than Fluo-4 during both Ca^2+^ release and SOCE phases. This difference was partly due to GCaMP’s expression in transfected cells only, as opposed to Fluo-4’s presence in the entire population. However, Fluo-4 also more accurately reported low Ca^2+^ concentrations and displayed a slightly faster dissociation rate (illustrated by the shape of SOCE peaks in Figure S5d and consistent with previous reports [72]). Nevertheless, two similar GCaMP fluorescence traces can be quantitatively compared within the relatively wide area of linear response. Therefore, despite its limitations, GCaMP was essential for our knockdown experiments, as it enabled specific recording of Ca^2+^ signals from transfected cells.

To determine the role of Orai2 in LTX^N4C^- and LPHN1-mediated [Ca^2+^]_cyt_ dynamics, differentiated LPH cells were co-transfected with the sh2 and GCaMP6S plasmids, and stimulated by TG, αLTX, or LTX^N4C^. GCaMP-Ca^2+^ fluorescence of knockdown cells was recorded in a microplate fluorometer. Orai2 knockdown did not affect Ca^2+^ release or influx under the basal (unstimulated) conditions (Figure S5e,f). However, the knockdown resulted in a 40–60% inhibition of both Ca^2+^ release and SOCE compared to control cells, upon stimulation with TG or αLTX (Figure 5b,c). This suggests that Orai2 mediated about 50% of SOCE in LPH cells, and the knockdown-induced decrease in Ca^2+^ influx could in turn affect the size of ER Ca^2+^ stores. Only the PostEq phase after TG stimulation did not differ in knockdown and control cells (Figure 5b), indicating that Ca^2+^_cyt_ extrusion machinery was not affected by Orai2 knockdown. LTX^N4C^-induced Ca^2+^ release and Ca^2+^ influx (SOCE/CICR) were also strongly decreased by Orai2 knockdown (Figure 5d).

In summary, while Orai2 contributes to the maintenance of Ca^2+^ stores and the influx stimulated by TG or latrotoxins, it is not critical, as its knockdown neither fully blocks SOCE nor depletes the stores. This indicates the involvement of other proteins in LPHN1-mediated Ca^2+^ dynamics. Indeed, we identified STIM2 as one such protein specifically upregulated by LPHN1, which aligns with our hypothesis that LTX^N4C^ acts by stimulating SOCE. We also reasoned that targeting STIM proteins could determine whether LPHN1 signaling opens Ca^2+^ channels directly or indirectly by store depletion and STIM activation. We therefore focused our subsequent investigation on STIM2.

### 2.6. The Role of STIM2 in LPHN1-Mediated LTX^N4C^ Action

The role of STIM2 in LPHN1-mediated LTX^N4C^ action was also studied using shRNA-based RNAi technology to knock down STIM2 expression in LPH cells. To alleviate the problems with the non-linearity of GCaMP fluorescence response, [Ca^2+^]_cyt_ recordings were again performed by loading the cells with Fluo-4 dye. However, lentivirus-mediated transduction of STIM2-targeting shRNA was used this time to overcome the low efficiency of plasmid transfection. Lentiviral transduction conditions, such as multiplicity of infection (MOI), cell density, duration of exposure, and polybrene concentration, were extensively optimized (Figure S6a,b). The transduction efficiency was quantified using the signal from red fluorescent protein (RFP), encoded by the lentiviral vector, and reached ~65% (Figure S6a,b).

**Figure 6:**
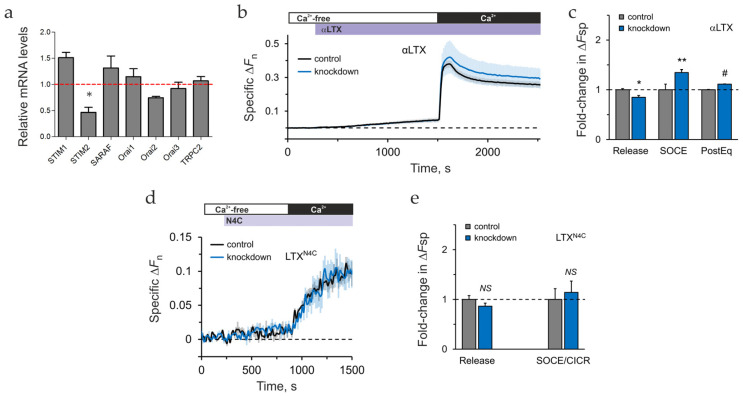
Knockdown of STIM2 mRNA downregulates Ca^2+^ stores but increases SOCE. (**a**–**e**) LPH cells were transduced with the lentivirus encoding anti-STIM2 shRNA at MOI = 60, allowed to grow for 24 h, differentiated in SF medium for 48 h, then loaded with Fluo-4 AM, and stimulated according to the standard protocol (shown above the traces), while recording the fluorescence response. (**a**) The effect of lentiviral shRNA transduction on mRNA levels of the main SOCE-associated proteins. The mRNA levels were determined using qRT-PCR and normalized to β-actin and control values for each protein (knockdown/control). (**b**–**e**) The effect of STIM2 knockdown on changes in Ca^2+^_cyt_ in response to stimulation with 1 nM αLTX or 3 nM LTX^N4C^. (**b**,**d**) Time-courses of specific (above background) changes in Ca^2+^_cyt_ fluorescence after normalization to baseline and subtraction of basal fluorescence. The dashed lines indicate 0. (**c**,**e**) Fold-change in aggregated (AUC) specific Ca^2+^ fluorescence in STIM2-knockdown cells relative to control cells during the Release, Influx/SOCE, and PostEq phases. For illustration, Δ*F*_n_ values were aggregated over time for each phase as AUCs. The dashed lines indicate the control level (fold-change 1). (**a**–**e**) The data are the means of *n* = 3 experiments performed in triplicates (*N* = 9) ± SE. Statistical tests applied were one-way ANOVA (**a**) and FANOVA (**c**,**e**). Asterisks denote statistical significance of the difference between knockdown and control cells for each condition; *, *p* < 0.05; **, *p* < 0.01; #, *p* < 0.0001; NS, non-significant.

The inhibition of STIM2 mRNA in transduced LPH cells was ascertained by qRT-PCR and constituted ~65% (Figure 6a), while other SOCE-associated proteins were not significantly affected. We then assessed the effect of STIM2 knockdown on Ca^2+^ signaling in cells loaded with Fluo-4 AM and stimulated with αLTX or LTX^N4C^ (Figure S6c–e and Figure 6b–e). Under basal conditions (no stimulation), Ca^2+^ release during incubation in Ca^2+^-free medium was slightly but insignificantly lower in knockdown cells compared to control LPH cells (Figure S6c,d). By contrast, Ca^2+^ influx was increased by ~28% in STIM2 knockdown LPH cells (Figure S6c,d).

STIM2 knockdown altered the Ca^2+^ response to αLTX stimulation (Figure 6b,c), resulting in a marginal reduction in specific (above background) Ca^2+^ influx, a substantial augmentation of specific SOCE amplitude, and a higher PostEq Ca^2+^ level compared to control cells. Finally, while STIM2 knockdown had no significant effect on LTX^N4C^-induced Ca^2+^ dynamics (Figure 6d), we noted a similar, although non-significant, trend toward decreased release and increased SOCE/CICR (Figure 6e).

These findings corroborate our data reported in Section 2.2, which identify the LTX^N4C^-sensitive Ca^2+^ pools as distinct from the ER, as they are not directly sensitive to TG. This non-ER nature of the toxin-mobilized stores indicates that the mutant toxin acts through a mechanism independent of the canonical ER-SOCC coupling pathway and thus does not involve STIM proteins. We discuss alternative mechanisms for Ca^2+^ release and influx in Section 2.8 and Section 3.6.

While the observation that STIM2 knockdown slightly reduces the size of Ca^2+^ stores but enhances SOCE seems counterintuitive, it is consistent with the established dual-role model of STIM proteins (discussed in Section 3.3). Importantly, the inability of STIM knockdown to affect LTX^N4C^ action excluded its involvement in LTX^N4C^-induced, LPHN1-mediated Ca^2+^ dynamics and indicated a direct signaling link between LPHN1 and Ca^2+^ channels.

Together, the Orai2 and STIM2 knockdown results ruled out SOCE as the main target of LPHN1-mediated signaling. Furthermore, the data described in Section 2.2 and Section 2.3 demonstrated that LTX^N4C^-induced Ca^2+^ spiking was always accompanied by a constant influx of Ca^2+^_e_, implicating the opening of specific Ca^2+^ channels. Therefore, we concentrated our subsequent efforts on identifying these Ca^2+^ channels.

### 2.7. VGCC Expression

While a large number of channels can provide Ca^2+^ entry pathways [73], one group potentially involved in LPHN1-mediated LTX^N4C^ actions in presynaptic nerve terminals is the VGCC family. While VGCCs are not part of the core SOCE mechanism, they can modulate it indirectly in excitable cells [74]. Importantly, VGCCs are central to CICR in myocytes/neurons by providing the initial Ca^2+^ spark [75,76] and thus could be functionally connected to LPHN1 signaling in synapses.

**Figure 7:**
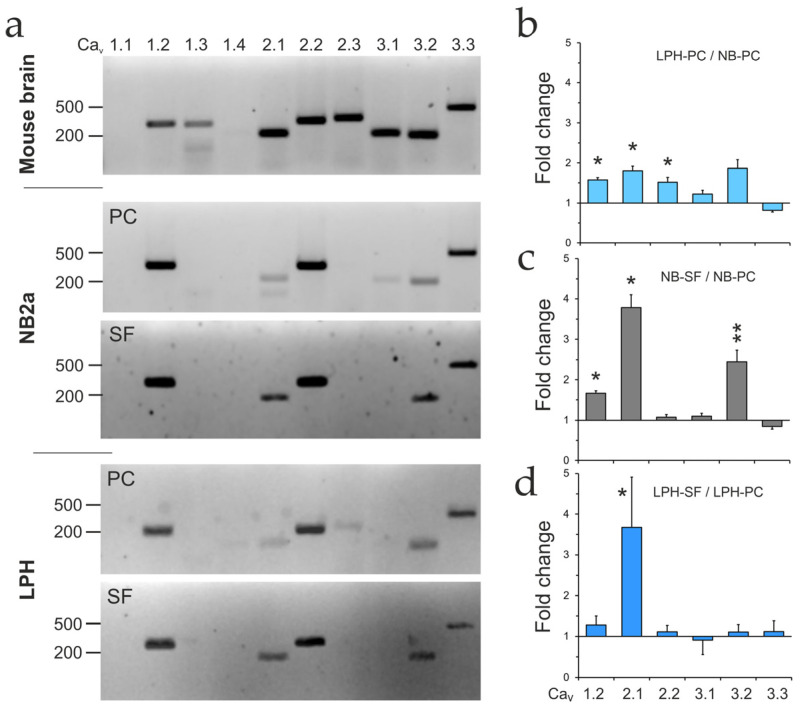
VGCC α1-subunits expression in proliferating and differentiated NB and LPH cells. NB and LPH cells were maintained in complete medium or differentiated in SF medium for 48 h. mRNA was isolated from mouse brain and the proliferating or differentiated cell cultures, reverse transcribed, and used to amplify the fragments of respective VGCC α1-subunit by qRT-PCR employing specific primers. (**a**) The amplification products were analyzed by agarose gel electrophoresis. Fragments of the following sizes (in bp) were expected: Ca_v_1.1 (334), Ca_v_1.2 (353), Ca_v_1.3 (337), Ca_v_1.4 (260), Ca_v_2.1 (253), Ca_v_2.2 (354), Ca_v_2.3 (376), Ca_v_3.1 (246), Ca_v_3.2 (230), Ca_v_3.3 (471). PC: proliferating cells; SF: cells differentiated in SF medium. Numbers on the left show the sizes of selected markers in bp. The gels are representative of *n* = 3 experiments with 3 replicates (*N* = 9), which gave similar results. (**b**) Fold changes in VGCC mRNA levels induced by receptor expression in proliferating LPH cells, relative to proliferating NB cells. (**c**) Fold changes in VGCC mRNA levels induced by SF differentiation of NB cells, relative to proliferating NB cells. (**d**) Fold changes in VGCC mRNA levels induced by SF differentiation of LPH cells, relative to proliferating LPH cells. In (**b**–**d**) the bars show the means ± SE from *n* = 3–4 independent experiments performed in triplicates (*N* = 9–12). Asterisks indicate statistical significance of differences between proliferating LPH and NB cells in (**b**), differentiated and proliferating NB cells in (**c**), and differentiated and proliferating LPH cells in (**d**). One-way ANOVA was applied to test statistics. Non-significant differences are not shown for simplicity; *, *p* < 0.05; **, *p* < 0.01.

Initially, the expression of different VGCC α1 subunits was assessed in NB and LPH cell lines, and compared to mouse brain using RT-PCR and agarose gel electrophoresis (Figure 7a). For all mRNAs tested, products of expected size were observed, confirming the specificity of the amplification reactions. All VGCC α1 subunits, except Ca_v_1.1 and 1.4, were detected in the mouse brain. Proliferating NB cells expressed only a subset of these VGCCs: one L-type channel (Ca_v_1.2), two neuronal channels: P/Q-type (Ca_v_2.1) and N-type (Ca_v_2.2), and all three T-type channels (Ca_v_3.1–3.3). However, in NB cells, the levels of Ca_v_2.1, 3.1, and 3.2 were significantly reduced, while Ca_v_1.3 and 2.3 were absent. Proliferating LPH cells expressed a similar repertoire of VGCCs as NB cells, but contained some Ca_v_2.3, while lacking Ca_v_3.1.

To reveal the effects of LPHN1 expression and cell differentiation on VGCC levels, we performed qRT-PCR in both proliferating and differentiating NB and LPH cells (Figure S7). For all mRNAs tested, the amplification and melting curves were consistent with specific amplification. As shown in Figure 7b, LPHN1 expression in proliferating cells upregulated all VGCC α1-subunits except Ca_v_3.3.

Differentiation in SF medium increased the levels of Ca_v_1.2, 2.1, and 3.2 in NB cells but only Ca_v_2.1 in LPH cells (Figure 7c,d). Notably, differentiation upregulated the neuronal channel Ca_v_2.1 especially strongly, corroborating our previous conclusion that it shifts NB cells toward a neuronal phenotype.

Based on these results, Ca_v_1.2 and Ca_v_2.1 were selected for further investigation of their role in LTX^N4C^ actions.

### 2.8. Ca_v_2.1 Is Critical for LPHN1-Mediated LTX^N4C^ Action

To assess the role of Ca_v_1.2 in LPHN1-mediated LTX^N4C^ action, we used specific L-type VGCC inhibitors [77]. Nimodipine is highly selective for L-type (Ca_v_1.1–1.3) calcium channels and does not interact with other Ca^2+^ channels or transporters (e.g., non-L-type VGCCs, Orai, TRPC channels) [77,78].

While Ca_v_2.1 (P/Q-type VGCC) is strongly upregulated in differentiated LPH cells, these cells also express much larger amounts of Ca_v_2.2 (N-type VGCC). Each of these channels can be inhibited separately, using specific blockers [77]. However, to determine the contribution of the whole group of neuronal VGCCs, we employed ω-conotoxin MVIIC, which blocks Ca_v_2.1 with high affinity and Ca_v_2.2 with lower affinity [79,80].

First, we determined the inhibitors’ effects on basal Ca^2+^ regulation and TG-induced SOCE and PostEq. Nimodipine and MVIIC were applied to LPH cells without stimulation and after stimulation with TG, when ER Ca^2+^ stores were depleted (Figure S8a). Under the basal conditions, MVIIC only marginally but significantly inhibited Ca^2+^ influx (Figure S8a,b). By contrast, nimodipine strongly reduced basal Ca^2+^ influx (Figure S8a–d). Consistent with the small impact of MVIIC, the effect of both inhibitors was not additive (Figure S8a,b). In TG-stimulated cells, only nimodipine reduced SOCE, but both inhibitors reduced the PostEq Ca^2+^ level (Figure S8a,c,d).

These results indicate that, in LPHN1-expressing cells, L-type VGCCs significantly contribute to both constitutive Ca^2+^ influx and TG-induced SOCE, whereas the neuronal VGCCs are only slightly involved in constitutive Ca^2+^ influx. Importantly, both types of VGCC must be open (or frequently opening and closing) at resting potential in these cells.

**Figure 8:**
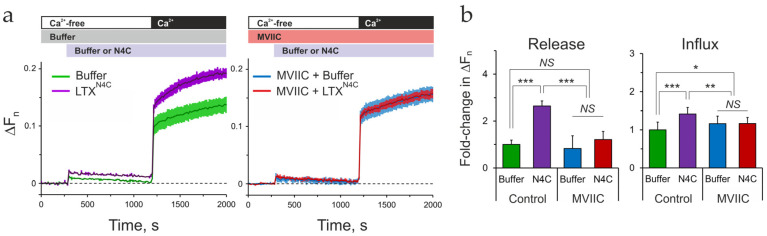
ω-Conotoxin MVIIC blocks LTX^N4C^-induced LPHN1-mediated SOCE/CICR. LPH-SF cells were incubated in Ca^2+^-free medium, exposed to 5 μM MVIIC or buffer (Control), then stimulated by 1 nM LTX^N4C^ or treated with buffer, and finally supplied with 2 mM Ca^2+^_e_, as shown by bar above. (**a**) Changes in Ca^2+^ fluorescence under basal conditions or induced by LTX^N4C^ in the absence (**left**) or presence (**right**) of MVIIC. The dashed lines represent zero. (**b**) Fold-changes in Ca^2+^ release (**left**) and Ca^2+^ influx (**right**) relative to buffer in the absence (Control) or presence of MIIC. The data are the means ± SE of *n* = 4 experiments (*N* = 12). Asterisks indicate the significance of differences (tested by FANOVA) between the connected values: *, *p* < 0.05; **, *p* < 0.01; ***, *p* < 0.001; NS, nonsignificant.

Subsequent experiments were conducted to assess whether nimodipine or MVIIC inhibits LPHN1-mediated increases in [Ca^2+^]_cyt_. LPH-SF cells were incubated in Ca^2+^-free buffer, exposed to MVIIC, then stimulated with LTX^N4C^ and supplied with Ca^2+^_e_ (Figure 8a,b). These experiments demonstrated that MVIIC inhibited LTX^N4C^-induced Ca^2+^ release and fully blocked the subsequent Ca^2+^ influx. In contrast to MVIIC, nimodipine did not inhibit but instead augmented LTX^N4C^-induced SOCE/CICR in LPH cells (Figure S8e).

These observations indicate that LTX^N4C^ stimulates the opening—or induces oscillations—of Ca_v_2.1 and/or Ca_v_2.2 channels in LPHN1-expressing cells, and that inhibiting these channels blocks LTX^N4C^ effects. Although LTX^N4C^-induced Ca^2+^ influx does not rely exclusively on Ca_v_2.1/2.2 activity and likely involves other mechanisms [56], these channels appear critical for specific components of LTX^N4C^-triggered Ca^2+^ influx. These include both the gradual Ca^2+^ rise and the Ca^2+^ spikes associated with combined SOCE and CICR. By contrast, Ca_v_1.2 opening does not contribute to LTX^N4C^-induced Ca^2+^ influx, which distinguishes its role from that in basal or TG-induced Ca^2+^ influx.

These findings demonstrate that LTX^N4C^ stimulates Ca_v_2.1/2.2 channels to open or oscillate in LPHN1-expressing cells, and that inhibiting these channels fully abrogates the LTX^N4C^-induced Ca^2+^ influx. While other mechanisms may contribute to LTX^N4C^ actions [56], Ca_v_2.1/2.2 are critical for generating both the gradual rise and the high-amplitude spikes of Ca^2+^ that characterize the LTX^N4C^ response. Conversely, Ca_v_1.2 is dispensable for the LPHN1-mediated pathway, while playing an important role in basal or TG-induced Ca^2+^ influx.

## 3. Discussion

### 3.1. NB Cells as a Neuronal Model

The present study establishes a role for LPHN1 in shaping calcium signaling dynamics, using NB cells as a model system. We demonstrate that expression of LPHN1, as well as chemical differentiation, promotes a shift toward a more neuronal morphology in these cells. To provide a foundational resource for interpreting Ca^2+^ signaling data in this model, we cataloged the expression of key proteins associated with SOCE and VGCCs. This provides a valuable resource for the community, profiling the signaling toolkit available in this commonly used neuronal model.

### 3.2. Deciphering the LTX^N4C^-Induced Calcium Signature

Our central findings demonstrate that the mutant toxin LTX^N4C^, which binds LPHN1 without pore formation, stimulates receptor signaling and evokes a complex Ca^2+^ response. This signaling was previously shown to involve a G protein cascade leading to PLC activation and IP_3_/DAG production [35,46,56]. The LTX^N4C^-induced Ca^2+^ response comprises Ca^2+^ release from intracellular stores during Ca^2+^-free incubation followed by pronounced influx upon Ca^2+^ re-addition. The mechanism connecting the signaling to Ca^2+^_cyt_ changes will be considered in Section 3.5, while here we will discuss the characteristics of the LTX^N4C^-induced Ca^2+^ signals.

We confirmed that in neuroblastoma cells, LTX^N4C^—similar to wild-type αLTX [45]—mobilizes Ca^2+^ pools distinct from TG-sensitive ER store, as mutant toxin and TG produce additive effects. A key feature of these pools is their ability to exchange Ca^2+^ with depleted ER. Pre-depleting the ER with TG reduces the amount of Ca^2+^ available for subsequent LTX^N4C^ release, whereas adding TG after the toxin allows Ca^2+^ release from both pools independently.

The depletion of these distinct stores triggers characteristically different Ca^2+^ influx pathways:

- Depletion of the LTXN4C-sensitive pools alone induces a gradual, non-inactivating influx, manifesting as combined asynchronous oscillations in individual cells.
- Depletion of the TG-sensitive ER alone produces a standard transient SOCE peak.
- When ER depletion follows LTXN4C-sensitive pools release, the subsequent SOCE is strongly augmented.
- Surprisingly, pre-depleting the ER, despite abolishing the LTXN4C-specific release, still produces an augmented SOCE.

This interplay suggests that LTX^N4C^ and TG not only target different stores but also activate Ca^2+^ influx through fundamentally different mechanisms. The LTX^N4C^-induced Ca^2+^ release is disproportionately small relative to the substantial, gradually developing influx it eventually triggers. Furthermore, the response is not an immediate influx through pre-activated channels, as seen with SOCE, but a delayed process that requires extracellular Ca^2+^ to fully develop.

We therefore propose a model wherein LPHN1 activation by LTX^N4C^ initiates a Ca^2+^_e_-dependent positive feedback loop. Ca^2+^_e_ acts as a critical co-factor that enters through an initial, limited number of channels, likely of a different type than SOCCs, and subsequently amplifies the signal to progressively activate a larger channel population, eventually leading to CICR and canonical SOCE.

These data partially confirm prior findings that LTX^N4C^-induced neurotransmitter release in neurons is Ca^2+^_e_-dependent and inhibited by TG [56]. However, our results demonstrate that in NB cells, ER depletion by TG inhibits LTX^N4C^-induced calcium release from internal pools but not Ca^2+^ influx from the extracellular space. This discrepancy may stem from morphological and functional differences between nerve terminals and NB cell bodies, the main target of LTX^N4C^ in our model expressing exogenous LPHN1. NB cells possess a larger cytosol and greater inter-organelle distances, where signaling processes such as IP_3_ diffusion and degradation could yield functionally distinct outcomes. The identity of the LTX^N4C^-sensitive, TG-insensitive calcium pools remains unknown, as does the mechanism of calcium exchange with the ER. These pools could represent specialized ER subregions or recyclable vesicular compartments that lack SERCA Ca^2+^ pumps, which warrants separate investigation.

Finally, a key insight emerged from comparing population-level fluorometry with single-cell Ca^2+^ imaging. While the population trace suggests a smooth, gradual Ca^2+^ rise, single-cell recordings revealed that LTX^N4C^, in fact, triggers asynchronous oscillatory Ca^2+^ events in individual cells. We interpret this as evidence for desynchronized SOCE, likely potentiated by CICR from ER stores. The summation of these high-amplitude but stochastic events across the population produces the averaged, smooth curve observed in bulk measurements. In addition, the single-cell Ca^2+^ imaging showed a constant, gradual increase in the background cytosolic Ca^2+^ concentration, likely representing a form of sustained Ca^2+^ influx. This feature provided an early indication that plasma membrane Ca^2+^ channels distinct from SOCCs might be involved.

### 3.3. Probing the Roles of Orai2 and STIM2

To dissect LPHN1-mediated signaling cascade, we employed RNAi against proteins upregulated by LPHN1 expression. This approach revealed that knockdown of Orai2 significantly attenuated the LTX^N4C^-induced calcium response.

Orai2 knockdown similarly affected Ca^2+^ dynamics evoked by three disparate stimulants, suggesting that Orai2 has a universal function. Therefore, we posit that Orai2, as a typical SOCC, likely contributes to the cell’s intrinsic capacity to sustain SOCE and associated CICR. Its knockdown probably reduces the overall amplitude of the cyclical Ca^2+^ spiking, thereby diminishing the population-averaged Ca^2+^ signal, but its removal was not critical for LTX^N4C^-induced Ca^2+^ influx. Thus, Orai2 appears to be important for the progression and amplification of the LTX^N4C^-induced signal, but not its initiation.

As the quantitative estimates of inhibition following Orai2 knockdown were derived from GCaMP fluorescence measurements, we needed to carefully consider key drawbacks of this sensor: non-linear response to [Ca^2+^], potentially variable expression in distinct cell lines, and finite Ca^2+^-binding kinetics. These technical difficulties were overcome in our experiments by utilizing the same cell line for knockdown and control, making all recordings under identical conditions, and ensuring that the observed calcium changes in both knockdown and control cells fell within the same dynamic, quasi-linear portion of GCaMP’s response. As our primary goal was not to measure absolute Ca^2+^ concentrations, but to identify a relative attenuation of the response in a targeted manner, GCaMP was ideally suited for this task due to its ability to be co-transfected with shRNA plasmids, ensuring that recorded signals originated exclusively from knockdown cells, a prerequisite for legitimate signal normalization.

Furthermore, the non-linearity of GCaMP’s response means that signals can be compressed at high Ca^2+^ concentrations, potentially underestimating the magnitude of SOCE in control cells. This suggests that the degree of SOCE inhibition may be even more pronounced than our estimates indicate. Therefore, our conservative reporting provides high confidence that Orai2 knockdown produces a significant and biologically relevant attenuation of the LTX^N4C^-induced calcium response.

Interestingly, knockdown of STIM2 revealed a more nuanced, regulatory role. We observed that STIM2 knockdown resulted in a slight decrease in agonist-induced ER Ca^2+^ release, yet paradoxically led to an enhancement of SOCE. This phenomenon can be explained by the distinct and often opposing roles of STIM1 and STIM2. STIM2 is known to form heteromultimers with STIM1 and act as a physiological “brake” on its more potent activity, helping to fine-tune the SOCE response [81,82,83]. The removal of STIM2 via knockdown likely releases this inhibition, leading to STIM1 hyperactivation, which, combined with STIM1’s compensatory overexpression (Figure 6a), amplifies SOCE, despite the reduced Ca^2+^ level in the ER. Furthermore, as a primary role of STIM2 is to maintain basal ER Ca^2+^ levels by triggering continuous, low-level SOCE, its knockdown can lower the “full” ER level because the stores are not being constantly refilled. However, this deregulation of both the stores and SOCE does not appear to play any role in the specific Ca^2+^ dynamics initiated by LTX^N4C^.

### 3.4. Identifying the Primary Ionic Effector: VGCCs

The characteristics of the LTX^N4C^-induced influx pointed directly to a channel-mediated process. When LTX^N4C^ is added in the presence of extracellular Ca^2+^, it gradually activates some channels and prepares Ca^2+^ stores for release, leading to bursts of Ca^2+^ after a certain delay, variable in individual cells. When applied in the absence of extracellular Ca^2+^, LTX^N4C^ pre-activates this system so that channels mediate a rapid Ca^2+^ influx immediately upon Ca^2+^e re-addition. These reactions occur with different efficiency in individual cells, producing the observed desynchronized spiking activity.

We initially considered receptor-operated calcium channels (ROCCs) as candidates. ROCCs, including the TRPC channel family, P2X receptor family and ionotropic neurotransmitter receptors, are gated directly by ligand binding or indirectly by intracellular signaling. However, LTX^N4C^ does not bind P2X or neurotransmitter receptors [36]. Among TRPC channels, the most common neuronal ROCCs, only TRPC2 is expressed in our model, and while it is upregulated by differentiation, its level is not affected by LPHN1 expression, making it an unlikely primary effector.

Crucially, the pharmacological profile of the LTX^N4C^-induced influx, described here, was definitive. The Ca^2+^ influx was completely abolished by ω-conotoxin MVIIC, a potent blocker of P/Q-type (Ca_v_2.1) and N-type (Ca_v_2.2) channels, but was unaffected by nimodipine, an L-type (Ca_v_1.2) channel blocker. Therefore, LTX^N4C^ specifically requires neuronal VGCCs (Ca_v_2.1/2.2) to initiate a downstream cascade that engages the cell’s own SOCE and CICR machinery.

Interestingly, we found that basal or TG-induced Ca^2+^ influx also involves VGCCs but has a distinct pharmacological profile, being nimodipine-sensitive and MVIIC-insensitive. The mechanism of this phenomenon likely includes membrane depolarization, the opening of Ca_v_1.2 channels, the entry of “trigger” Ca^2+^, and the induction of CICR, which then leads to SOCE.

### 3.5. A Model for LPHN1-Mediated Activation of Neuronal VGCCs

The requirement for VGCCs in LTX^N4C^- or TG-induced Ca^2+^ influx presents some mechanistic questions:

(i) Can VGCCs function in LPH cells, which do not maintain a high membrane potential? The resting potential of differentiated NB cells (−40 to −55 mV) is sufficiently negative to prevent spontaneous VGCC activation but permissive for depolarization-induced opening. At this potential, while some P/Q and N-type channels are inactivated, a significant portion of these channels are in a closed but available state, poised to open upon depolarization. This biophysical setting is ideal for generating the oscillatory Ca^2+^ activity we observed.

L-type (Ca_v_1.2) channels, the most abundant VGCCs in LPH cells, under these conditions will be primarily in a closed, but available state, because their activation threshold is near −40 mV, and they are known for their highly negative voltage-dependence of inactivation. A cell resting at −40 mV has most of its L-type channels ready to be opened by any further depolarization. These considerations suggest that both Ca_v_1.2 and Ca_v_2.1/2.2 will be able to contribute to Ca^2+^ influx upon membrane depolarization.

(ii) What is the mechanism by which LPHN1 activation leads to the opening of Ca_v_2.1/2.2 channels, which are normally gated by membrane depolarization? We propose several non-exclusive mechanisms for how LPHN1 could activate VGCCs:

1. Receptor-induced membrane depolarization: Gαq-coupled GPCRs can depolarize the membrane by inhibiting potassium channels (e.g., M-current channels) or activating non-selective cation channels. In fact, LTX^N4C^ has been shown to induce insulin exocytosis in pancreatic β cells by acting via LPHN1 to inhibit voltage-gated K^+^ channels, which was followed by Ca^2+^ transients [58]. However, in our current model, LTX^N4C^ is unlikely to act via membrane depolarization, as this should have also engaged Ca_v_1.2.

2. Channel modulation by second messengers: LPHN1 activates a Gαq/PLCβ pathway [35,56], generating DAG and IP_3_. The DAG–PKC axis is involved in a well-established mechanism for an indirect VGCC potentiation. For instance, Cav2.1 and Cav2.2 channels are known to be potentiated by PKC phosphorylation downstream of Gq-coupled receptor signaling and DAG generation, which reduces voltage-dependent inactivation, thereby prolonging the open state and facilitating sustained Ca^2+^ entry. While direct evidence for any kinase activation by LTX^N4C^ is currently lacking, the receptor’s CTF itself undergoes activity-dependent phosphorylation/dephosphorylation, a process that modulates the CTF-NTF interaction. Specifically, LTX binding to the NTF has been shown to induce CTF dephosphorylation and its dissociation from the NTF [84]. This post-translational modification could potentially alter the CTF’s interactions with downstream signaling proteins or associated VGCCs. More generally, this finding indicates that specific kinases and phosphatases are co-localized with LPHN1 in neurons and thus are positioned to directly regulate VGCCs that may be recruited to the vicinity of the activated receptor, for instance, via the “toxin bridge” (Mechanism 3).

Furthermore, DAG itself can directly gate a subset of ion channels, notably specific TRPC channels (e.g., TRPC3, C6, C7), causing them to open and allow cation influx (including Ca^2+^), which can lead to membrane depolarization.

Although these second messenger and phosphorylation signaling pathways are likely engaged by LTX^N4C^ and could contribute to its effects, they do not directly explain the toxin’s selectivity for Cav2.x and therefore warrant specific future investigation.

3. Direct protein–protein interaction (“toxin bridge”): LTX^N4C^ could act as a physical bridge, simultaneously binding to LPHN1 and a specific subunit of a VGCC. The “toxin bridge” model provides a potential structural basis for the observed Cav2.x selectivity. In this scenario, LTX^N4C^’s high-affinity interaction with LPHN1 positions it to make a second, specific contact with an extracellular loop unique to Cav2.1/2.2 channels, but not Cav1.x. This selective bridging would not only localize the toxin to the correct channel but also allosterically gate it, stabilizing the channel in an open conformation and thus effectively lowering its voltage-dependent activation threshold and facilitating Ca^2+^ influx. For the “toxin bridge” to be feasible, LPHN1 must be localized in the plasma membrane close to VGCCs. With receptor overexpression in NB cells, such a co-localization is entirely plausible. However, targeted studies are required to explore this possibility in neurons.

4. Signalosome complex formation: LPHN1, its associated signaling proteins (like G-proteins), and VGCCs may be organized within a signaling complex, ensuring highly efficient and specific channel regulation. This model represents an evolution of mechanism 3 above. The characteristic lag phase before LTX^N4C^ action is consistent with a signaling mechanism that may involve the gradual accumulation of a critical second messenger or the complex assembly. This priming phase ultimately sets the stage for the rapid, VGCC-dependent initiation of asynchronous SOCE/CICR oscillations upon Ca^2+^_e_ re-addition. Importantly, a signaling complex could specifically recruit selected VGCCs.

It must be noted, however, that this hypothesis does not exclude, but can incorporate any of the alternative mechanisms listed above. Moreover, although the signalosome concept can explain many, if not all, of LTX^N4C^’s actions via LPHN1, there is currently little direct evidence for signalosome formation. Therefore, the precise mechanism by which LPHN1-mediated signaling activates VGCCs remains a subject for future investigation.

### 3.6. Uniform Priming and Asynchronous Ca^2+^ Spiking

A key intriguing finding of this work is the lack of synchronized channel opening in a system uniformly primed by LTX^N4C^ via LPHN1. The following discussion proposes a mechanism for this phenomenon, supported by our experimental observations and existing literature.

LTX^N4C^–LPHN1 signaling via the Gαq–PLC pathway produces a relatively low level of IP_3_ [46], which binds to and primes the IP_3_ receptor (an ER-resident Ca^2+^ release channel) but does not cause it to open fully. The IP_3_ receptor is known to require two coincident signals—IP_3_ and a local Ca^2+^ rise—to open fully [85]. Although LTX^N4C^ stimulates Ca^2+^ release from non-ER pools, the resulting [Ca^2+^]cyt is much smaller than that induced by TG (Figure 2 and Section 3.3) and is likely insufficient to support the opening of IP_3_ receptors and store depletion. Thus, in contrast to TG, LTX^N4C^ signaling only primes the IP_3_ receptor and fails to activate STIM1/2 and the subsequent opening of SOCCs in the plasma membrane. Critically, however, LTX^N4C^ also activates Cav2.x channels in the plasma membrane. Therefore, when Ca^2+^ is added, it does not flood the cell via SOCCs to produce the characteristic SOCE peak. Instead, Ca^2+^ enters via a limited number of LTX^N4C^-activated Cav2.x channels, as revealed by our observations. This slow, localized increase in [Ca^2+^]cyt creates a positive feedback loop, amplifying the Ca^2+^ signal until it is sufficient to gate the primed IP_3_ receptors and trigger massive Ca^2+^ release (CICR). The resulting store depletion then leads to SOCE, and the concerted action of CICR and SOCE [73,85] produces the Ca^2+^ spikes (Figure 3). These spikes represent an intrinsic cellular mechanism of Ca^2+^ signaling and homeostasis, with LTX^N4C^ only acting as a trigger.

The fundamental explanation of the desynchronized response in cells ready for a massive Ca^2+^ influx lies in the probabilistic and cell-to-cell variable nature of the final triggering event. The process of LPHN1 signaling, IP_3_ receptor priming, and slow Ca^2+^ influx via VGCCs depends on several cell-specific factors, such as the level of LPHN1 expression, membrane potential, and the number and localization of VGCCs or IP_3_ receptors. As a result, each cell reaches the CICR threshold at a stochastically determined time, leading to asynchronous Ca^2+^ oscillations driven by the subsequent cell-specific CICR/SOCE interplay. This elegantly explains how a uniform priming stimulus results in the observed asynchronous sharp oscillations at the single-cell level but a gradual kinetic trace at the population level.

### 3.7. Limitations and Future Directions

While NB cells display many neuronal features, especially upon expression of LPHN1 and differentiation, they can only be employed as a first-approximation model in deciphering the physiological effects in nerve terminals. They do not form proper synapses, so presynaptic events are modeled over the cell body, a much larger environment with different spatial constraints. Furthermore, NB cells express a slightly altered repertoire of signaling proteins compared to neurons, and LPHN1 overexpression may lead to non-native interactions.

However, despite the anatomical and functional differences between nerve terminals and NB cells, our finding that TG indirectly affects LTX^N4C^-sensitive Ca^2+^ stores in NB cells is partially in line with the previous observation that TG inhibits LTX^N4C^-induced glutamate release in hippocampal neurons [56]. Moreover, LTX^N4C^ has been shown to stimulate bursts of neurotransmitter release in motor neuron nerve terminals by acting via LPHN1 [50,86], which fits very well with the high-amplitude bursts of [Ca^2+^]_cyt_ induced in NB cells expressing the full-size receptor only (Figure 3 and Figure S3). These data confirm that NB cells are a vital, accessible surrogate system that allows for genetic manipulation and reduces animal use.

Nevertheless, given the many limitations of NB cells as a neuronal model, it will be necessary to validate these findings in neurons. Among the specific questions that need to be addressed are (i) the identity of LTX^N4C^-sensitive Ca^2+^ stores and their relationship with ER, (ii) the mechanism by which LPHN1-mediated signaling activates Ca_v_2.1/2.2, and (iii) the specific type(s) of VGCC involved.

## 4. Materials and Methods

### 4.1. Materials

All materials were from Sigma-Aldrich (Sigma-Aldrich Company Ltd., Gillingham, Dorset, UK) unless otherwise stated. The LPH and ΔLPH constructs were generated by subcloning the cDNA encoding the full-size rat LPHN1 or its NTF and the cDNA encoding a C-terminal fragment of rat neurexin Iα into the pcDNA3.1 vector, downstream of the pCMV promoter, as described earlier [87].

### 4.2. Cell Culture

The generation of stably transfected cell lines was described in [35]. NB cells were cultured in DMEM containing GlutaMAX^TM^ and 10% fetal bovine serum (FBS). Stably transfected NB cell lines were always maintained in 300 μg/mL G418. Cells were allowed to grow at 37 °C and 5% CO_2_ to 80% confluency before passaging (every 2–3 days). For differentiation, 24 h after plating, the cells were washed with phosphate-buffered saline (PBS), and the FBS medium was replaced with SF-medium (Neurobasal-A medium containing 2% B-27 supplement, 0.5 mM GlutaMAX^TM^, and 1 mM dbcAMP when required). Cells were differentiated for 24–48 h and until 70–80% confluent.

### 4.3. RNA Extraction

Total RNA from respective cells was isolated using a High Pure RNA Isolation Kit (Roche Products Limited, Welwyn Garden City, UK). Total RNA from mouse spinal cord and the ventral horn of its lumbar segment were isolated as follows. Spinal column dissected from a 21–28-day-old mouse was purchased from Charles River Laboratories (Margate, UK). The spinal cord was ejected from the isolated sacral region of the spinal column by hydrostatic pressure exerted using a P200 pipette tip attached to a syringe filled with PBS. Whole spinal cord was homogenized in lysis buffer (E.Z.N.A. Total RNA Kit 1, Omega Bio-tek, Avantor, Inc., Lutterworth, UK) with a Potter-Elvehjem homogenizer (Sartorius, Epsom, UK) or cut into 1 mm sections using a McIlwain tissue chopper (Campden Instruments, Loughborough, UK). The sections were incubated in chilled oxygenated artificial cerebrospinal fluid (in mM: 119, NaCl; 26.2, NaHCO_3_; 2.5, KCl; 1, NaH_2_PO_4_; 2.5, CaCl_2_; 1.3, MgCl_2_, 10; glucose). The ventral horn was dissected from each segment and put directly into lysis buffer. Total RNA was isolated using an E.Z.N.A. Total RNA Kit 1 (Omega Bio-tek).

### 4.4. RT-PCR

cDNA was synthesized using 1 μg total RNA and anchored-oligo(dT) 18 primers using the Transcriptor First Strand cDNA Synthesis Kit (Roche Products Limited). Amplification of cDNA targets was performed using Taq Polymerase (Fermentas UK Limited, Cambridge, UK). The reaction mix (50 μL) included (μL): 1, cDNA (1:10 dilution); 0.25, Taq Polymerase (final concentration: 1.25 U per 50 μL reaction); 5, 10X Fermentas Reaction buffer with Mg^2+^ (final concentration 1.5 mM); 1, 10 mM dNTPs; 1, 10 μM forward and reverse primers; 40.75, nuclease-free water. RT-PCR reactions consisted of an initial denaturation step of 3 min 30 s at 95 °C, followed by 30 cycles comprising the following steps: 45 s at 95 °C, 45 s at 52–60 °C, and 45 s at 72 °C. Reactions were completed with a final elongation step of 5 min at 72 °C. Primers (Table 1 and Table 2) were designed using Lasergene 9.1.1 software suite (DNASTAR, Inc., Madison, WI, USA), so that the products crossed exon-exon junctions to control genomic DNA amplification. PCR products were analyzed by 2% agarose gel electrophoresis, stained with ethidium bromide, and imaged at 16-bit depth (to ensure a high dynamic range) using a G:Box gel documentation system (Syngene, Cambridge, UK). Product sizes were estimated by comparison to Generuler 50 bp DNA ladder (Thermo-Fisher Scientific—UK, Life Technologies Limited, Dartford, UK).

### 4.5. Quantification of mRNA Expression

qRT-PCR for relative quantification of expressed genes was performed on LightCycler 480 (Roche Products Limited) using SYBR Green I Master reaction mix (Roche Products Limited) and the specific primers described in Section 4.4. Reactions consisted of a 5 min denaturation at 95 °C, followed by 40–45 cycles including: 10 s at 95 °C, 20 s at 52–60 °C, and 10 s at 72 °C. A final elongation step included 5 min at 72 °C. Product fluorescence was measured at one point of each cycle (80 °C). A melting curve recording was performed from 65 to 97 °C (2.2 °C/s) with continuous detection. Amplification of correct products was confirmed by using the LightCycler melting temperature (*T*_m_) analysis and by agarose gel electrophoresis. To demonstrate that only cDNA template was amplified, a no-template control (NTC) reaction was included for all targets, in which cDNA was replaced with nuclease-free water.

Raw fluorescence data was analyzed using LinRegPCR quantitative PCR data analysis program [88]. The initial amount of target cDNA (*N*_0_) in a sample (in AFU) was determined using Equation (1). For each individual reaction the baseline fluorescence was determined and then subtracted from the fluorescence curve. Then LinRegPCR calculated the PCR efficiency for each reaction. For each target group, a fluorescence threshold (*N*_t_) was set and the mean reaction efficiency (*E*_mean_) was used to convert the number of cycles, at which a reaction passes the threshold (quantification cycle, *C*q), to the initial concentration (*N*_0_). Differentiated samples were normalized to proliferating samples to determine fold-changes in mRNA levels.

(1) $$N_{0}=N_{t}/E_{mean}^{C_{q}}$$

Two housekeeping genes (β-actin cyclophilin-D) were used as reference genes. Expression levels of these genes consistently correlated, which indicates their suitability as reference genes. The presence of residual genomic DNA (gDNA) was assessed by qRT-PCR on all samples using 1 μL of undiluted total RNA, using primers targeting β-actin. The level of gDNA was extremely low and did not affect relative quantification.

### 4.6. Immunocytochemistry

Cells grown on 13 mm poly-D-lysine-coated glass coverslips were washed with ice-cold PBS (3 × 5 min), fixed with 400 μL 4% paraformaldehyde (*v*/*v*) for 10 min at room temperature, and washed with PBS (3 × 5 min). The cells were permeabilized with 0.1% (*v*/*v*) Triton X-100 for 7 min, then washed with PBS (3 × 5 min) and blocked in 5% (*v*/*v*) goat serum for 1 h at room temperature. Antibodies were diluted in 5% (*v*/*v*) goat serum. The cells were incubated with primary antibodies by placing the coverslips, cell side down, on top of 30 μL antibody solution spotted on a sheet of Parafilm™, for 1 h, washed (3 × 5 min), incubated with secondary antibodies for 1 h, and washed (3 × 5 min). The cells were then incubated with 0.3 μM DAPI for 5 min, washed (3 × 5 min), mounted onto glass slides, and sealed with nail varnish and stored at 4 °C.

For co-localization of LPHN1 fragments with lysosomes, LPH-SF cells were incubated with 100 nM LysoTracker™ Deep Red (Thermo-Fisher Scientific) for 30 min at 37 °C, then washed, fixed, permeabilized, and immunostained as described above.

The following antibodies were used: anti-V5 polyclonal rabbit IgG (1:1000 dilution) (Merck Life Science UK Limited, Gillingham, Dorset, UK); anti-myc mouse monoclonal antibodies (1:1000) (Bio-Rad Laboratories Limited, Watford, UK); anti-NF-H rabbit IgG (1:1000) (Neuromics, Minneapolis, MN, USA); anti-mouse Alexa Fluor-568-conjugated goat IgG (1:2000) and anti-rabbit Alexa Fluor-488-conjugated goat IgG (1:2000) (Molecular Probes, Thermo-Fisher Scientific).

Confocal microscopy of immunostained cells was carried out as described previously [50]. Briefly, cell preparations were imaged using a DMI8-CS laser scanning confocal microscope (Leica Mikrosysteme Vertrieb GmbH, Wetzlar, Germany) equipped with a 63×/1.4 oil immersion objective. The following configuration was used in double-staining experiments: laser excitation at 488 and 561 nm; emission filters 521 ± 31 and 608 ± 31 nm. For triple-staining, the settings also included excitation at 633 nm and emission detection at >650 nm. Images for subcellular distribution studies were obtained by scanning 1-μm-thick confocal sections near the cell’s equator. Laser power and detector gain were kept constant for all samples to allow for quantitative comparison, except when image over-exposure was required to demonstrate low levels of LPHN1 expression. Composite RGB images (1024 × 1024 pixels) were saved as TIFF files in the Leica Application Suite software LAS X 2.0.0.14332 (Leica Mikrosysteme Vertrieb GmbH, Wetzlar, Germany).

### 4.7. Fluorescent Ca^2+^_cyt_ Recordings

#### 4.7.1. Loading Cells with Fluo-4 AM

Cells were seeded onto 96-well clear-bottomed, black-walled plates (Agilent Technologies LDA UK Limited, Stockport, UK) at a density of 5000 cells per well, in DMEM containing GlutaMAX™ and 10% FBS, then differentiated as described above. Experiments were performed in a recording buffer (RB) consisting of (in mM): 145, NaCl; 5.6, KCl; 5.6, glucose; 1, MgCl_2_; 15, HEPES; 0.25, sulfinpyrazone; 0.5 mg/mL BSA 0.5 mg/mL. Cells were loaded with cell-permeant Ca^2+^-indicator Fluo-4 AM immediately before experiments. For this purpose, 50 μg Fluo-4 AM was dissolved in 20 μL DMSO containing 10% Pluronic F-127™ and then diluted with Neurobasal-A medium to a final concentration of 2 μM (the final concentration of Pluronic F-127™ was less than 0.01%). This solution was added to the cells and incubated for 20 min at 37 °C, protected from light. The cells were then washed twice and incubated for a further 20 min at 37 °C to allow for dye de-esterification.

#### 4.7.2. Population-Level Ca^2+^_cyt_ Fluorescence Recording with Fluo-4

Fluorescent measurements were made on a Fluoroskan Ascent FL microplate fluorometer (Labsystems Diagnostics Oy, Vantaa, Finland), using 485/538 nm excitation/emission filters and excitation beam diameter of 3 mm. Fluorescent intensity was measured every 15 s, with 100 ms integration time. The cells were maintained at 25 °C. Baseline fluorescence was measured for 180–300 s in Ca^2+^-free RB (stage 1). Pharmacological compounds and toxins were added to individual wells by pipette (stage 2) and RB containing Ca^2+^ (final concentration: 2 mM) was added by an automatic dispenser (stage 3). Maximum fluorescence was determined using 0.1% Triton X-100 to permeabilize all cells or with 1 nM αLTX, acting as a Ca^2+^ ionophore to permeabilize LPHN1-expressing cells only (stage 4). Initial volume in each well was 50–75 μL and compounds were added in 10–25 μL. Total volume at the end of experiment ranged from 100 to 150 μL. Experiments were usually performed in triplicates and repeated independently at least three times.

Fluorescence values *F* were normalized to the average baseline value (*F*_0_) and the average maximal value achieved with αLTX or Triton X-100 permeabilization (*F*_max_). Changes in Ca^2+^_cyt_ are reported as changes in normalized fluorescence (Δ*F*_n_) (Equation (2)).

(2) $${∆F}_{n}=\frac{F-F_{0}}{F_{max}-F_{0}}$$

Characteristic Ca^2+^ dynamics were quantified as the AUC of Δ*F*_n_ above the baseline for the three key phases of the experiment: Ca^2+^ release was measured following a stimulus application during the Ca^2+^-free phase. SOCE was measured above the Ca^2+^ influx line defined as the Ca^2+^ trend between the release phase and PostEq level. PostEq Ca^2+^ level was measured just before permeabilization.

The [Ca^2+^]_cyt_ during LTX^N4C^-induced Ca^2+^ spikes was estimated using Equation (3) [89,90], where *K*d is the dissociation constant of Fluo-4 for Ca^2+^ (770 nM at 25 °C) and *F*_min_ is Fluo-4 fluorescence in the presence of the Ca^2+^ chelator EGTA (200 μM) [45]:

(3) $${\left[{Ca}^{2+}\right]}_{cyt}=K_{d}\times \left(\frac{F-F_{min}}{F_{max}-F}\right)$$

LPHN1-mediated Ca^2+^ signaling was triggered using αLTX or LTX^N4C^. In αLTX experiments Ca^2+^ release, SOCE, and PostEq were quantified as in TG experiments. LTX^N4C^ action caused a gradual increase in Ca^2+^ influx, which was measured as Ca^2+^ influx above basal prior to the PosEq plateau. αLTX was used to obtain *F*_max_ in receptor-expressing cells only.

#### 4.7.3. Ca^2+^_cyt_ Fluorescence Recording in Single Cells by Confocal Microscopy with Fluo-4

Cells were plated on poly-D-lysine-coated coverslips placed inside 30 mm Petri dishes or 6-well plates, differentiated, and loaded with Fluo-4 AM, as described above. The medium was then replaced with RB, and the cells were imaged using a laser-scanning upright microscope Axioplan 2 LSM510 (Zeiss UK, Cambridge, UK) equipped with a water-dipping objective (Achroplan, 40×, Zeiss UK). Two-dimensional confocal images were taken every 5 s, using 488 nm laser excitation and a 505–550 nm emission filter. Time-series images were converted into fluorescence changes over time using LSM510 software AxioVision 4.9.1 SP2 (Zeiss UK). Fluorescence was integrated within regions of interest (ROIs) drawn around individual cells or neurite varicosities. The fluorescence traces were normalized to *F*_0_ and *F*_max_, as described by Equation (2).

#### 4.7.4. Ca^2+^_cyt_ Recording with GCaMP

GCaMP6S was used to record from cells simultaneously transfected with more than one plasmid. LPH cells were co-transfected with the GCaMP6S plasmid and one of the shRNA plasmids, as described in Section 4.9. After 24 h, the cells were washed with PBS and differentiated in SF medium. Ca^2+^ recordings were performed after 72 h, using the population-level Fluo-4 protocol above, except the integration time was 300 ms. Fluorescence values were normalized, reported, and quantified according to the same method.

### 4.8. Plasmid-Mediated Knockdown

A set of four plasmids encoding shRNAs targeting mouse Orai2 mRNA was purchased from Dharmacon, Inc. (Waltham, MA, USA). shRNAs in the pLKO.1 lentiviral vector contained the following mature antisense sequences: TTAGACCCTTATTCATGCGGG (TRCN0000126314; sh1); ATGAGCAGAGCAAACAGATGC (TRCN0000126315; sh2); TAATCCATGCCCTTGTGGCCG (TRCN0000126317; sh3); TACCATGATGATGGTGGACAC (TRCN0000126318; sh4). The shRNA plasmids were amplified in DH5α bacterial cells and purified using QIAGEN^®^ Plasmid Midi kit (QIAGEN Ltd., Manchester, UK) and introduced into the target cells by transfection as described in Section 4.9.

### 4.9. Plasmid Transfection

To evaluate Orai2 knockdown produced by each shRNA construct, LPH cells were seeded in 6-well plates at 1.5 × 10^5^ cells/well 24 h prior to transfection. Four μg of each shRNA plasmid was diluted in 400 μL DMEM containing 6 μL Turbofect (Thermo-Fisher Scientific), mixed, incubated for 20 min at room temperature, and slowly added to the cells. After 24 h, the cells were washed with PBS and differentiated in SF medium for 72 h. The cells were harvested and used to isolate RNA for quantification of the Orai2 and other SOCE mRNA levels by qRT-PCR (Section 4.3, Section 4.4 and Section 4.5).

For transient co-transfection with the shRNA and GCaMP6S plasmids, LPH cells were seeded into 96-well plates (5000 cells/well) 24 h prior to transfection. For each well, 110 ng plasmid of interest and 90 ng of the GCaMP6S plasmid were diluted in 20 μL DMEM containing 0.3 μL Turbofect (Thermo-Fisher Scientific), mixed by vortexing, incubated for 20 min at room temperature, and added to the cells dropwise. Control cells were transfected with GCaMP6S only.

### 4.10. Knocdown by Lentiviral Vector Transduction

SMARTvector™ Lentiviral vector encoding STIM2-targeting shRNA and RFP as a reference protein was purchased from Dharmacon, Inc. in the form of purified lentiviral particles (clone ID V3SVMM08_10955358; 100 μL; 10^8^ transduction units/mL). Subsequent procedures were conducted in accordance with the manufacturer’s instructions. Firstly, an optimization experiment was carried out in a 96-well plate to determine the optimal lentiviral transduction conditions, including cell density, duration of exposure to reduced medium volume, and Polybrene concentration. Cells were seeded at three densities (3000, 4000, and 5000 cells/well), grown for 24 h, and exposed to a range of Polybrene concentrations (0, 2, 4, 8, 12, 16, 20, or 24 μg/mL) in reduced medium volume (50 μL of DMEM containing 10% FBS) for 6 h or 16–20 h. 24 h after incubation under this range of conditions, the medium was replaced with SF medium, and cell confluence was monitored for 24–72 h. The optimal conditions (confluence of 70–80%), achieved after a 6 h exposure to 4 μg/mL Polybrene and a starting cell density of 5000 cells/well, were used for subsequent lentiviral transduction experiments. Secondly, a range of virus/cell ratios (multiplicity of infection, MOI) was tested, and the MOI of 60 was subsequently used, consistent with the known refractoriness of neuroblastoma cells to lentiviral infection (Technical Manual, Dharmacon, Inc.).

For the knockdown experiments, LPH cells were seeded into 96-well plates at 5000 cells/well, allowed to grow in complete medium for 24 h, after which the medium was replaced with 50 μL of DMEM with 10% serum containing 4 μg/mL Polybrene and different dilutions of the lentivirus particles, and incubated for 6 h. One hundred μL of complete medium was then added, and the cells were allowed to grow for 24 h, when the medium was replaced with SF medium to induce cell differentiation. After another 48 h incubation, the lentivirus-transduced and differentiated cells were loaded with the Fluo-4 AM dye as described in Section 4.7.1 and used in Ca^2+^_cyt_ measurement experiments.

### 4.11. Western Blotting

Western blotting was performed as described previously [35]. Briefly, LPH-SF and LPH-PC cells were washed, solubilized in 1% Triton X-100, and analyzed by SDS-electrophoresis in 10% polyacrylamide gels and Western blotting. The samples were prepared by heating for 30 min at 50 °C in a conventional SDS buffer. Separated proteins were transferred onto Immobilon^®^-P membrane (Merck Life Science UK Limited, Gillingham, Dorset, UK) employing a wet electro-transfer unit (Bio-Rad Laboratories Limited). Protein bands were visualized using primary antibodies (the anti-NTF rabbit serum RL1 and anti-CTF rabbit serum R4 [91]), horseradish peroxidase-conjugated anti-rabbit goat IgG (Sigma-Aldrich) as secondary antibodies, and a chemiluminescent substrate (Merck). Luminescent signals were captured with a LAS-3000 Fujifilm gel imager (Raytek Scientific Ltd., Sheffield, UK).

### 4.12. Image Analysis

Images of RT-PCR products separated by agarose gel electrophoresis, taken at 16-bit depth, were used for illustration purposes only, while quantitative analysis was solely based on the results of qRT-PCR (Section 4.5).

Time-series confocal images of Fluo-4 fluorescence in live cells were converted into Δ*F*_n_ traces by normalization to *F*_0_ and *F*_max_ (see Equation (2)). *F*_max_ was obtained after permeabilizing LPH or ΔLPH cells with αLTX. Normalized traces were subsequently quantified as outlined in Section 4.7.2.

Confocal fluorescent images of fixed immunostained cells were used to quantify the subcellular distribution of LPHN1 fragments and their colocalization with lysosomes. The images were preprocessed by background removal and deconvolution and analyzed using ImageJ software (Fiji distribution, version 1.54g) (Laboratory for Optical and Computational Instrumentation, Madison, WI, USA). The preprocessed images of individual cells were then segmented into the plasma membrane and cytoplasm and masked. The NTF and CTF fluorescent signals were integrated for each segment and normalized to the total fluorescent signal.

The NTF and CTF colocalization with lysosomes was quantified by pixel intensity correlation analysis using the Coloc 2 plugin in the Fiji suite. To assess the degree of colocalization, Pearson’s correlation coefficient *r* and Manders’ split colocalization coefficients (M1 and M2) were used.

Images were prepared for illustrations using ImageJ (Fiji distribution). Any post-acquisition adjustments (e.g., color inversion or changes to contrast) necessary for visualization were applied across the entire image.

### 4.13. Data Analysis

Data were analyzed in R 3.3.0 (R Foundation for Statistical Computing, Vienna, Austria) and MS Excel (Microsoft Corporation, Redmond, WA, USA). Data are generally presented as mean ± SE of *n* determinations. Statistical analysis was performed in Prism 8.02 software (GraphPad Software, Boston, MA, USA). The Lilliefors test was applied to determine whether datasets followed a normal distribution. Unless otherwise stated, the two-tailed Student’s *t*-test was typically performed for comparisons between two groups with equal variances; otherwise, the nonparametric Mann–Whitney test was applied. One-way analysis of variance (ANOVA) with Bonferroni correction was used for three or more groups. Three-way ANOVA and Tukey–Kramer post hoc test were applied to analyze neurite outgrowth in N- and S-type cells. To determine the statistical significance of the differences between curves, an FDA [63] was performed in R 3.3.0. Its results were verified by a spreadsheet-based modified Chi-squared method for comparing arbitrary curves [92]. For graphical presentation of quantitative differences, the curves were represented by respective AUC values. The statistical significance was accepted at *p* < 0.05; the level of significance was indicated on graphs (*, *p* < 0.05; **, *p* < 0.01; ***, *p* < 0.001; #, *p* < 0.0001; *NS*, non-significant).

## Figures and Tables

**Table 1:** Primers used to amplify SOCE-associated mRNA and reference mRNA.

Target	Primer Sequence	Size (bp)	Annealing T *, °C	
	(Forward/Reverse)		Optimal	Used
STIM1	CCGCCCTAACCCCGCCCACT/CCCCCTCAATCAGCCGATGGC	296	62.1	60
STIM2	TCAGCCGGCAATGATAGCAAG/TGGAAAGCCCCAGTGGAGTTA	256	54.6	55
SARAF	GCGCCTCCTCCGGGCTTTAA/TCCCTGCGCCTCCACCCA	280	61.4	60
Orai1	CGGGACGCTGCTTTTCCTA/CGGTGTTAGAGAATGGTCCCC	335	61.2	60
Orai2	CCTGTGGCCCCCAGATGTTGA/AGTACTGGCCCCCACGCAAGC	327	59.9	60
Orai3	ACAGACCGCCACAAGCAGGAG/GCAGGCGGGCCTCTTTCC	318	59.4	55
TRPC1	GAATCGCGTAACCAGCTCAGC/CTGCAGTGGGCCCAAAATAGA	225	55.2	55
TRPC2	AAGGCCGCAGCCAGAGTGTCT/AGGAGGCGCAGTGCAAAGGAT	181	58.3	60
TRPC3	GGAGGGGCCCCGGGAGTACAT/TCCGGGAGAAGCTGAGCACCA	284	59.8	60
TRPC4	TTTGTTGGGGCCACCATGTTT/CGCCCAATTGTCCCGAAGC	299	55.5	55
TRPC5	AAAACAAATGAGGGGCTAACA/CTTGGGCGCCACTAGCTCTTG	280	54.4	55
TRPC6	CTCAAGGCCCCAAAGAATACT/GTCCCCCAGTGTGACTTTTGT	179	51.8	55
TRPC7	GGCCGCGGGAGTACGTGCTA/CAACCGCAATGGCGTACAGCC	261	60.3	60
β-actin	TTCGCGGGCGACGATGC/GGGGCCACACGCAGCTCATT	233	60.2	60
Cycl. **	TAAGCATGATCGGGAGGGTT/CGTCCAGATGAGGAGTCGGAA	101	52.9	55

* T, temperature. ** Cycl., cyclophilin D.

**Table 2:** Primers used to amplify VGCC α1-subunit mRNA.

Target	Primer Sequence	Size (bp)	Annealing T *, °C	
	(Forward/Reverse)		Optimal	Used
Ca_v_1.1	ACGCCAATGCCAATGTT/ACGTGCTCCTCAAAGTTCC	334	56.4	56
Ca_v_1.2	CAGACCCCTACGGCCCATCCCTACCCTA/TGTCTGCGGCGTTCTCCATCTCCTCTATTG	353	64.0	63
Ca_v_1.3	CGCGCTGCCCTGCCCCTG/CACTCCTCTGCTTGTCGCTGTTCTTGTTC	337	62.0	61
Ca_v_1.4	ACCATGTGCCACGCCGACG/GCCGCCAAGTTTGCCAAGGTATCC	260	61.1	61
Ca_v_2.1	CAAAGCCCGGCGACTGGATGACTACTC/GTGGTGGTGGTGGTGTGGCCGATGCTTCC	253	63.4	63
Ca_v_2.2	GACCCCACGCCCCAGCATCACCTACAAGA/CCATTGGGTACACGGCGGAGA	354	61.7	61
Ca_v_2.3	GCCACCAAAGCCTCGTCCCCTCCTCTCC/CCTCCGCCGCCGATAGTGCCCGTTAG	376	65.2	63
Ca_v_3.1	GGCGCCATCCCTAAACTACC/CAGGCGGATGTGCTTGGAGACTTT	246	60.5	61
Ca_v_3.2	CCCGGCCGATGAGGAGGTC/GGCCATCCCCATTATCCAGTTCC	230	61.5	61
Ca_v_3.3	GGGGGCCATTCCATTCAACC/GCCCGCAGCCCACGCAGACTA	471	62.4	63

* T, temperature.

## Data Availability

The original contributions presented in this study are included in the article/Supplementary Material. Further inquiries can be directed to the corresponding author.

## Supplementary Materials

The following supporting information can be downloaded at: https://www.mdpi.com/article/10.3390/ijms262211200/s1.

## Abbreviations

The following abbreviations are used in this manuscript:

Abbreviation	Explanation
[Ca^2+^]	Ca^2+^ concentration
ADGRL1	Adhesion G-protein-coupled receptor L-type 1
AFU	Arbitrary fluorescence units
AGPCR	Adhesion GPCR
AM	Acetoxymethyl ester
AUC	Area under the curve
Ca^2+^_cyt_	Cytosolic Ca^2+^
Ca^2+^_e_	Extracellular Ca^2+^
Ca_v_	α-Subunit of a VGCC
CICR	Ca^2+^-induced Ca^2+^ release
CTF	C-terminal fragment
DAG	Diacylglycerol
dbcAMP	Dibutyryl cAMP
ER	Endoplasmic reticulum
FDA	Functional data analysis
GPCR	G-protein-coupled receptor
IP_3_	Inositol 1,4,5-trisphosphate
KD	Knockdown
LPH-dbcA	NB cells stably expressing LPHN1 and differentiated by dbcAMP
LPHN1	Latrophilin 1
LPH-PC	Proliferating NB cells stably transfected with LPHN1
LPH-SF	NB cells stably expressing LPHN1 and differentiated by serum deprivation
MOI	Multiplicity of infection
NB	Neuroblastoma 2a
NRX	Neurexin I
NTF	N-terminal fragment
PBS	Phosphate-buffered saline
PIP_2_	Phosphatidylinositol 4,5-bisphosphate
PKC	Protein kinase C
PLC	Phospholipase C
PostEq	Post-SOCE Ca^2+^_cyt_ equilibrium
qRT-PCR	Quantitative RT-PCR
RB	Recording buffer
RFP	(Turbo) Red fluorescent protein
RFU	Relative fluorescence units
ROCC	Receptor-operated Ca^2+^ channel
ROI	Region of interest
RT-PCR	Reverse-transcription polymerase chain reaction
RyR	Ryanodine receptor, Ca^2+^ release channel
SARAF	SOCE-associated regulatory factor
SDS	Sodium dodecyl sulfate
SERCA	Sarcoplasmic/endoplasmic reticulum Ca^2+^-ATPase (Ca^2+^ pump)
SF	Serum-free
shRNA	Small hairpin RNA
SOCC	Store-operated Ca^2+^ channel
SOCE	Store-operated Ca^2+^ entry
STIM	Stromal interaction molecules
TG	Thapsigargin
TRPC	Transient receptor potential canonical
VGCC	Voltage-gated Ca^2+^ channel
αLTX	α-Latrotoxin
ΔLPH-dbcA	NB cells stably expressing ΔLPHN and differentiated by dbcAMP
ΔLPH-PC	Proliferating NB cells stably transfected with ΔLPHN
ΔLPH-SF	NB cells stably expressing ΔLPHN and differentiated by serum deprivation
