# Supplementary material for: The Mechanism of LTXN4C-Induced Ca2+ Influx Involves Latrophilin-Mediated Activation of Cav2.x Channels
Source: Int J Mol Sci. 2025 Nov 19;26(22):11200. doi: 10.3390/ijms262211200 (PMC12653939; doi:10.3390/ijms262211200)
Supplement: Supplementary file 1 [file ijms-26-11200-s001.zip › ijms-3981819-supplementary.pdf]

International Journal of
Molecular Sciences

MDPI

Supplementary material

The Mechanism of LTX^{N4C}-induced Ca^{2+} Influx Involves Latrophilin-mediated Activation of $Ca_V2.x$ Channels

Jennifer K. Blackburn, John-Paul Silva, Evelina Petitto, Dietmar Cholewa, Elizaveta Fasler-Kan, Kirill E. Volynski, and Yuri A. Ushkaryov

For the quantification of neuronal phenotype, the following parameters were chosen: cell body shape (Figure 1b, c), the number of neurites per cell (Figure S1a), and the length of the longest neurite (Figure S1b). We also determined the percentage of cells in which neurites were longer than 50 µm, a threshold used as a differentiation marker to indicate a neuronal phenotype [49] (Figure S1c). Also, the subcellular distribution and degradation of LPHN1 in differentiated and proliferating LPH cells was studied (Figure S2d, e).

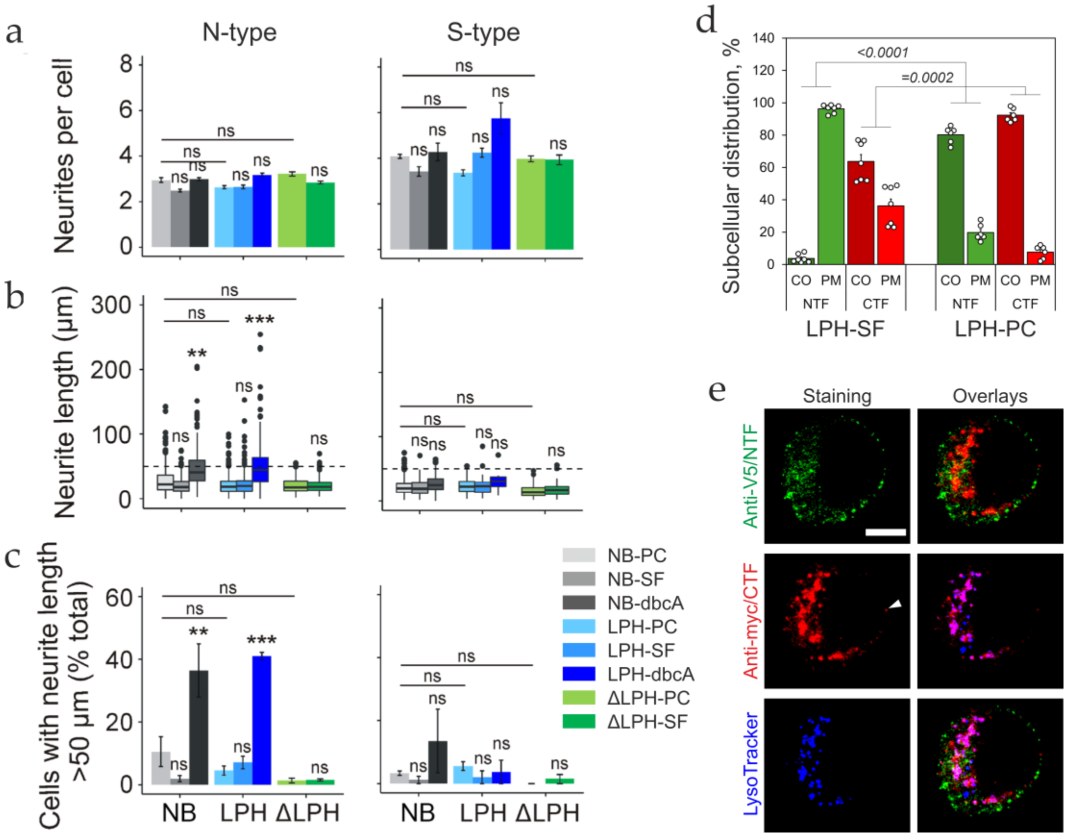

Figure S1. Differentiation-induced morphological changes in NB and receptor-expressing cells and receptor degradation in proliferating LPH cells. Cells were differentiated by growing for 48 h in SF medium ± 1 mM dbcAMP. Neurites of N- and S-type cells were measured separately. (**a**) Average number of neurites per cell. (**b**) The length of the longest neurite in each cell. (**c**) Percent of cells with a neurite longer than 50 µm. Three repeat cultures (n = 3) were analyzed for each cell line and differentiation treatment. 201–392 N-type cells and 12–277 S-type cells were analyzed per culture. PC, proliferating cells; SF, cells differentiated by serum deprivation; dbcA, cells differentiated by serum deprivation with 1 mM dbcAMP. The bars show the means (median in **b**) ± SE. Asterisks above bars show statistical significance (assessed by three-way ANOVA) compared to respective PC cells;

horizontal lines compare proliferating receptor-expressing cells to NB-PC cells; **, $p < 0.01$; ***, $p < 0.001$; *NS*, non-significant. (**d**) Relative subcellular distribution of the NTF and CTF in differentiated (SF) and proliferating (PC) LPH cells. CO, cytoplasmic organelles; PM, plasma membrane. The data are the means ± SE from $n = 6–7$ experiments, with $N = 112$ and 154 cells analyzed for each condition. Statistical significance of the differences (p, using one-way ANOVA) between the connected pairs of value is indicated above respective lines. (**e**) LPH degradation in proliferating LPH cells. Confocal images of an LPH-PC cell immunostained with an anti-V5 antibody for the NTF (green) and an anti-myc antibody for the CTF (red). Lysosomes were identified using the LysoTracker dye (false colored blue). Note that the CTF primarily concentrates in lysosomes, with only small amounts present in the plasma membrane (arrowhead). Scale bar, 5 μm.

To quantify Ca^{2+} dynamics induced by LTX^{N4C}, a standard protocol for measuring Ca^{2+} release from stores and SOCE was adopted. Figure S2a demonstrates the changes in Ca^{2+}_{cyt} levels stimulated by TG, αLTX, and LTX^{N4C} in LPH-SF cells, and defines respective quantification parameters. To confirm the specificity of LTX^{N4C} actions, control experiments were conducted using proliferating LPH cells and differentiated ΔLPH cells (Figure S2b, c).

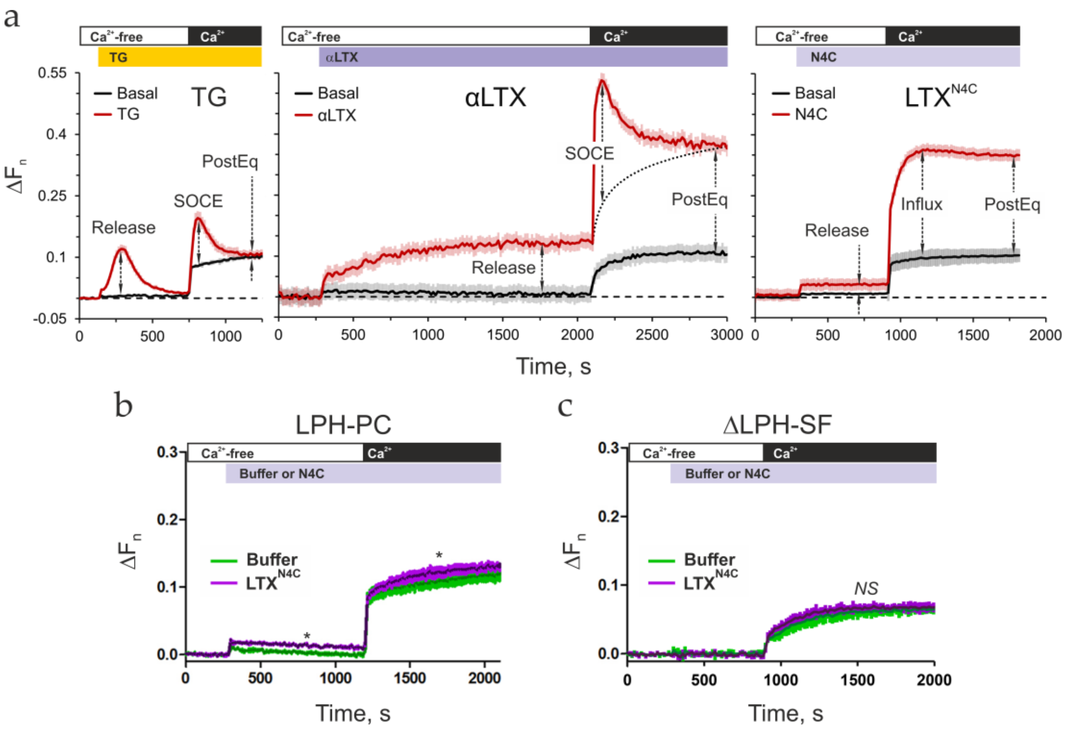

Figure S2. Approaches to quantifying Ca^{2+} dynamics and control experiments. (**a**) Determination of Ca^{2+}_{cyt} levels during the Release, SOCE and PostEq phases under different stimulation conditions. LPH cells were differentiated in SF medium, loaded with Fluo-4 AM, and stimulated with 0.3 μM TG (left), 1 nM αLTX (middle), or 3 nM LTX^{N4C} (right), at the times indicated above the graphs. As the mechanism of the Ca^{2+} influx induced by LTX^{N4C} is unknown, it is identified as a generic Influx rather than SOCE. (**b, c**) LTX^{N4C}-induced changes in Ca^{2+}_{cyt} levels in LPH-PC cells and ΔLPH-SF cells. The cells were allowed to grow in complete medium (LPH-PC) or differentiated for 48 h in SF medium (ΔLPH-SF) and loaded with Fluo-4 AM. The graphs show time courses of Ca^{2+} fluorescence changes in LPH-PC cells (**b**) or LPH-SF cells (**c**) treated with buffer or 1 nM LTX^{N4C}. All traces are normalized to F_0 and F_{max}, as detailed in Section 4.7. The data are the means of $n = 3$ experiments ± SD. Statistical significance of the differences between control and stimulated traces are shown above respective curves: *, $p = 0.038$, FANOVA; NS, non-significant, $p = 0.19$, FANOVA. Note that LTX^{N4C} triggers a small Ca^{2+} influx in proliferating cells expressing the full-size LPHN1 only.

To ascertain the role of receptor in LTX^{N4C}-induced changes in Ca^{2+}_{cyt} levels, Ca^{2+} fluorescence was recorded in individual ΔLPH-expressing cells stimulated with LTX^{N4C}. (Figure S3).

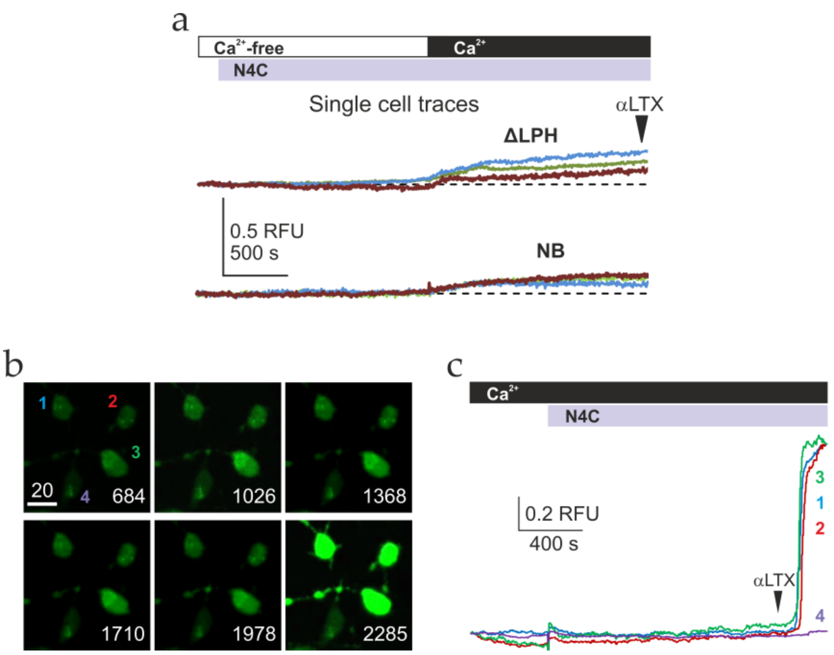

Figure S3. LTX^{N4C} does not affect Ca^{2+}_{cyt} levels in ΔLPH and NB cells. (**a**) Individual normalized fluorescent traces ± SE from selected ΔLPH and NB cells, treated as described in Figure 3a. RFU, normalized relative fluorescence units. (**b**) Selected time-lapsed fluorescent images of a group of cells (1–4), which were treated as described in Figure 3c. The numbers indicate the time in seconds from the beginning of recording; the scale bar is 20 μm. (**c**) Time course of fluorescence intensity changes in individual cells from (**b**). Relative fluorescence intensity (ΔF_n) was normalized to the maximal fluorescence F_{max} revealed by αLTX pore formation. LTX^{N4C} and αLTX additions are shown by arrowheads. The numbers indicate corresponding cells in (**b**). Note that cells 1–3 (expressing ΔLPH) did not respond to LTX^{N4C} but showed strong Ca^{2+} fluorescence when permeabilized by αLTX, while cell 4 (not expressing the receptor) did not respond to either toxin. The experiment is a representative of n = 9 independent experiments, with 4 replicates (N = 36), which showed similar results.

The levels of mRNA for SOCE-associated proteins were tested by qRT-PCR in proliferating and differentiated NB cells and receptor-expressing cells (Figure S4).

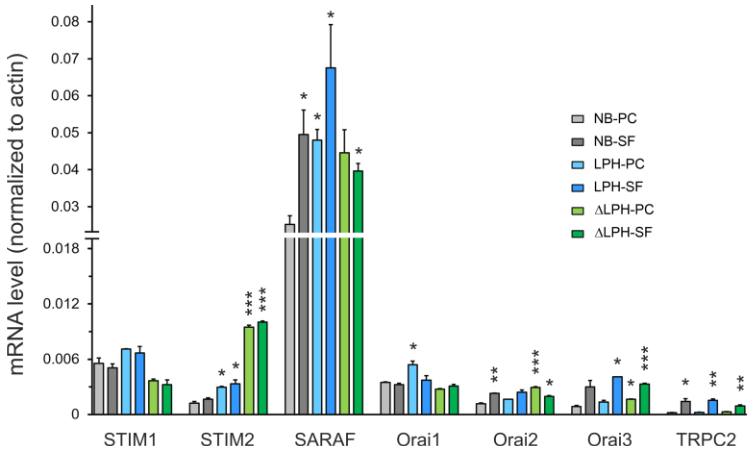

Figure S4. Expression of SOCE-associated proteins in proliferating and differentiated NB, LPH, and ΔLPH cells. qRT-PCR was performed on cDNA produced from NB cells (NB) and two stably

transfected cell lines (LPH and ΔLPH) that were proliferating (PC) or differentiated in SF medium (SF). The SOCE protein mRNA levels were normalized to actin. The bars show the means ± SE (n = 3–4) with 3 replicates (N = 9–12). Asterisks above bars denote statistical significance (assessed by one-way ANOVA) compared to proliferating NB cells (NB-PC) for each protein; *, $p < 0.05$; **, $p < 0.01$; ***, $p < 0.001$; the non-significant differences are not indicated for simplicity.

Figure S5 describes the results of experiments conducted to (i) select shRNA(s) for Orai2 knockdown, (ii) compare the fluorescent responses of Fluo-4 and GCaMP, and (iii) assess the effect of Orai2 knockdown on basal Ca^{2+} influx.

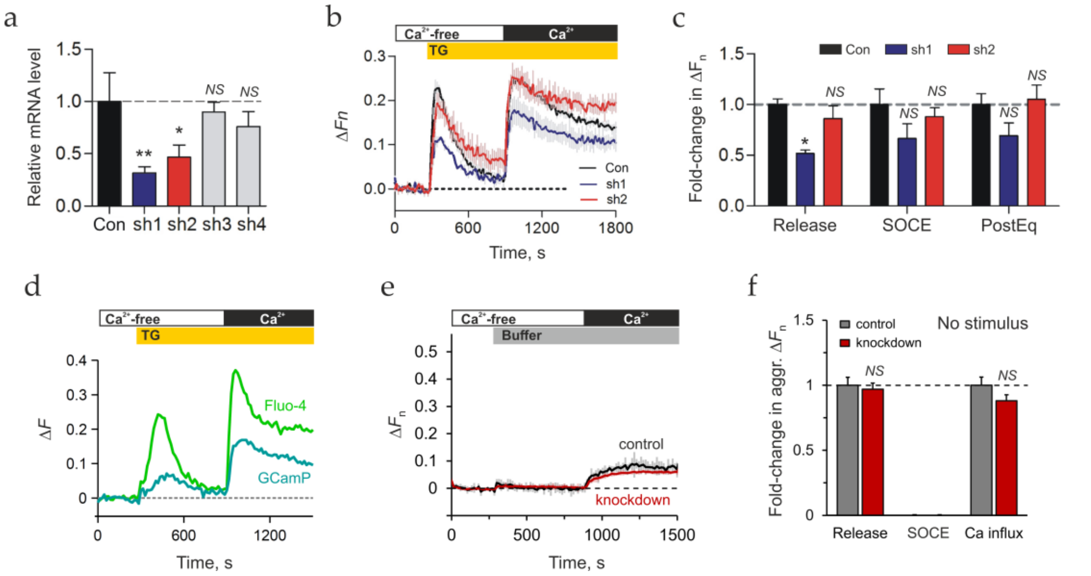

Figure S5. Selection of shRNAs for Orai2 knockdown and Ca^{2+}_{cyt} detection using the expression of a Ca^{2+}-sensing protein, GCaMP. (**a–f**) Prior to fluorescence recordings, LPH or NB cells were transfected with a plasmid encoding one of the shRNAs (sh1–sh4), a plasmid encoding GCaMP, or both an shRNA and the GCaMP plasmid. The cells were then allowed to grow for 24 h and differentiated in SF medium for 48 h. (**a**) The levels of Orai2 mRNA in LPH cells transfected with individual shRNA plasmids. The mRNA levels were quantified by qRT-PCR and normalized to β-actin and untransfected control cells (Con). (**b**) The effect of transfection with the sh1 or sh2 plasmid on TG-induced store depletion and Ca^{2+} influx in the overall cell population. Ca^{2+}_{cyt} fluorescence was recorded in control (untransfected) and shRNA-transfected NB cells, which were loaded with Fluo-4 AM and exposed to 0.3 µM TG and 2 mM Ca^{2+}_{e} (as shown by the bars above). Averaged ΔF_n traces are shown. (**c**) Changes in Ca^{2+}_{cyt} levels in sh1- and sh2-transfected cells relative to control (untransfected) cells during the Release, SOCE, and Ca^{2+} Equilibrium phases. The data are from several experiments as in (**b**). (**d**) The use of GCaMP to detect changes in Ca^{2+}_{cyt} in transfected cells only. Changes in Ca^{2+}_{cyt} fluorescence (ΔF) induced by 0.3 µM TG were recorded in control NB cells loaded with Fluo-4 AM and NB cells transfected with GCaMP. Note that GCaMP-transfected cultures exhibit a robust response to changes in Ca^{2+}cyt, although GCaMP is expressed in a subset of cells only. (**e, f**) The effect of Orai2-knockdown on changes in Ca^{2+}_{cyt} levels under basal conditions (with no stimulus applied). Ca^{2+}_{cyt} fluorescence was recorded in LPH cells expressing GCaMP alone (control) or both GCaMP and sh2 (knockdown). (**e**) Averaged normalized Ca^{2+} fluorescence traces (ΔF_n) in control and Orai2 knockdown cells under basal conditions (as shown by the bars above). (**f**) Fold-changes in aggregated Ca^{2+} fluorescence in knockdown cells relative to control cells during the indicated phases, as in (**e**). For comparison, ΔF_n values were aggregated over time for each phase as AUCs. (**a-f**) The data are the means of n = 3 experiments with 3 replicates (N = 9) ± SD (**a–c**) or SE (**e, f**). One-way ANOVA (**a, c**) and FANOVA (**f**) were applied. Asterisks denote statistical significance relative to control cells; *, $p < 0.05$; **, $p < 0.01$; NS, non-significant.

The apparent incomplete Orai2 knockdown in Figure S5a is likely the result of measuring the mRNA level in the entire heterogeneous cell population. The shRNA-mediated knockdown was only effective in the fraction of cells that were successfully transfected, while the untransfected cells maintained normal mRNA levels, thus diluting the observed effect.

Figure S6 describes the results of experiments to (i) optimize the multiplicity of lentiviral transduction in LPH cells, (ii) determine the effects of STIM2 knockdown on basal Ca^{2+} influx, and (iii) assess changes in Ca^{2+} fluorescence upon cells' treatment with buffer, LTX^{N4C}, and αLTX.

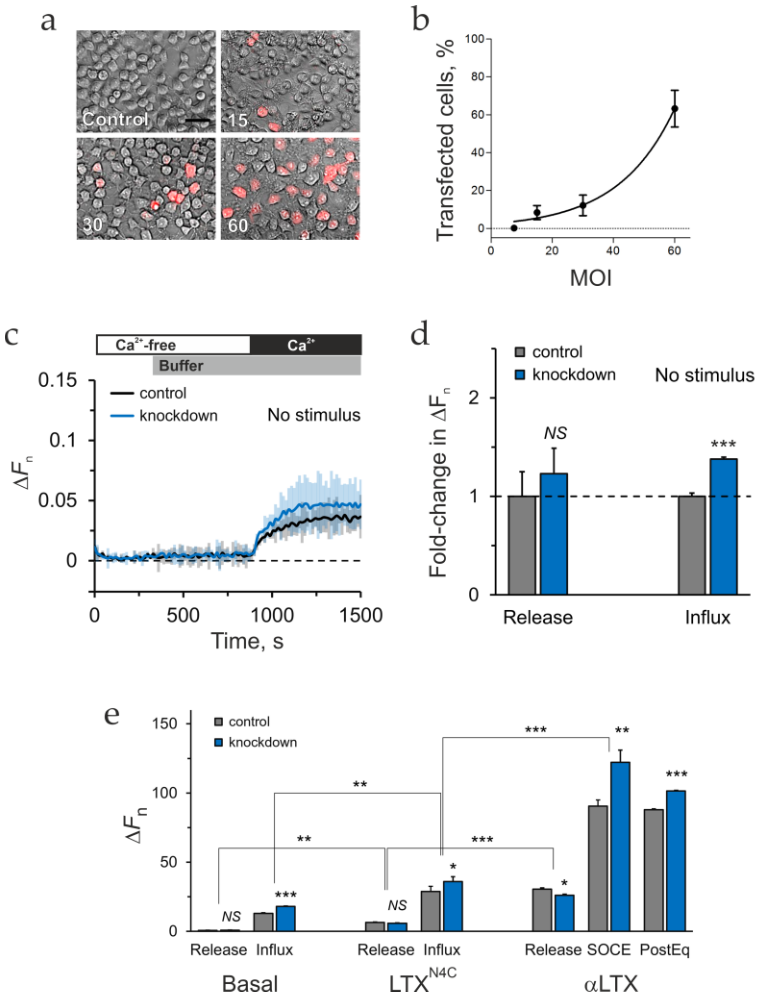

Figure S6. Optimization of conditions for STIM2 knockdown. (**a,b**) Optimization of multiplicity of infection (MOI). LPH cells were exposed to lentiviral particles at varying MOIs as described in methods, allowed to grow for 24 h, then differentiated in SF medium for 48 h. (**a**) Composite (fluorescent and phase-contrast) images of cells exposed to viral particles at different MOIs, as indicated. Lentivirus-transduced cells are identified by red fluorescence due to RFP expression. Scale bar, 50 µm. (**b**) Percentage of cells expressing RFP after transduction. (**c-e**) The effect of STIM2-knockdown on changes in Ca^{2+}_{cyt} levels. LPH cells were transduced with STIM2-targeting lentiviral particles at MOI = 60, loaded with Fluo-4 AM, and stimulated according to the standard protocol (shown above the traces), while recording the fluorescence response. (**c**) Averaged normalized traces of changes in Ca^{2+} fluorescence (ΔF_n) in control and knockdown cultures under basal conditions (no stimulus applied). (**d**) Fold-changes in aggregated Ca^{2+} fluorescence during the Release and Influx phases under basal conditions, relative to control cells. (**e**) Average normalized changes in Ca^{2+}_{cyt} fluorescence in control and STIM2 knockdown LPH cells during the indicated phases (Release, Influx/SOCE, and

PostEq), under different stimulation conditions: Basal (no stimulus), 3 nM LTX^{N4C}, or 1 nM αLTX. (**d,e**) For illustration purposes, ΔF_n values were aggregated over time for each phase as AUCs. (**b-e**) The data are the means ± SE from $n = 3$ experiments done in triplicates ($N = 9$). Symbols denote statistical significance (tested by FANOVA) relative to control cells or as shown by lines; *, $p < 0.05$; **, $p < 0.01$; ***, $p > 0.001$; NS, non-significant.

qRT-PCR was used to assess the changes in the expression of mRNA for VGCCs caused by cell differentiation and expression of the LPHN1 constructs (Figure S7).

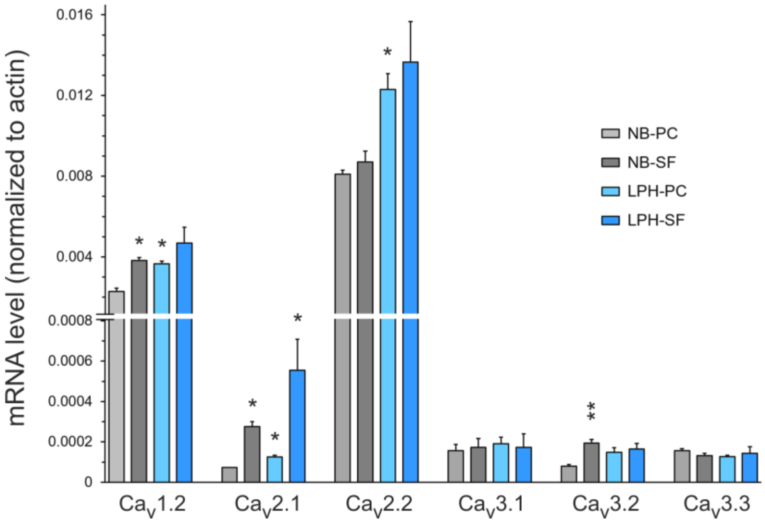

Figure S7. Expression of VGCC α1-subunits in proliferating and differentiated NB and LPH cells. mRNA was isolated from NB cells (NB) and LPH cells which were proliferating (PC) or differentiated in SF medium (SF), reverse transcribed, and used to amplify the fragments of respective VGCC α1-subunit by qRT-PCR employing specific primers. The mRNA levels were then normalized to that of β-actin. The bars are the means ± SE from $n = 3–4$ independent experiments done in triplicates ($N = 9–12$). For each protein, asterisks denote statistical significance of differences (assessed by one-way ANOVA) between proliferating NB cells (NB-PC) and the other conditions (NB-SF, LPH-PC, and LPH-SF); non-significant differences are not indicated for simplicity; *, $p < 0.05$; **, $p < 0.01$.

Nimodipine (an inhibitor of L-type Cav) and ω-conotoxin MVIIC (a blocker of P/Q- and N-type Cav) were used to determine the role of Cav1.2 and Cav2.1/2.2, respectively, in the effects of TG and LTX^{N4C} on Ca^{2+} release and influx in NB and LPH cells (Figure S8).

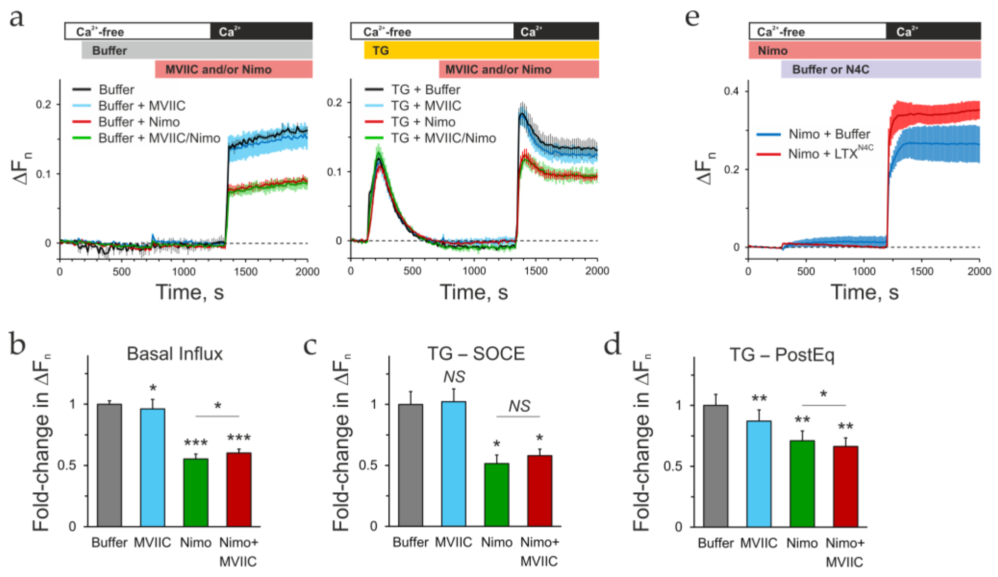

Figure S8. Nimodipine inhibits both the basal and TG-induced Ca^{2+} influx but does not affect LTX^{N4C} action. (**a**) VGCCs of the L-type, but not P/Q- or N-type, contribute to basal Ca^{2+} influx and TG-induced SOCE. LPH-SF cells were incubated in Ca^{2+}-free buffer containing 0.1 mM EGTA, treated with buffer (**left**) or stimulated 0.3 μM TG (**right**), exposed to 1 μM MVIIC and/or 10 μM nimodipine (Nimo), and finally supplied with 2 mM Ca^{2+}_e, as shown by the bars above. (**b**) Fold-changes in the amplitude of Ca^{2+} influx relative to control (no Cav inhibitors) in unstimulated cells. (**c, d**) Same as (**b**) but in TG-stimulated cells: (**c**) SOCE; (**d**) PostEq. (**e**) L-type VGCCs do not contribute to LTX^{N4C}-induced SOCE/CICR. LPH-SF cells were incubated in a Ca^{2+}-free buffer, stimulated with 1 nM LTX^{N4C}, then exposed to 10 μM nimodipine (Nimo), and supplied with 2 mM Ca^{2+}_e. The traces are representative of $n = 4$ independent experiments, showing the mean ± SE of four replicates; the histograms are the means ± SE of n = 4 experiments ($N = 16$). Asterisks denote statistical significance of differences (tested by FANOVA and one-way ANOVA) from control or as indicated by lines: *, $p < 0.05$; **, $p < 0.01$; ***, $p > 0.001$; NS, non-significant.

Disclaimer/Publisher's Note: The statements, opinions and data contained in all publications are solely those of the individual author(s) and contributor(s) and not of MDPI and/or the editor(s). MDPI and/or the editor(s) disclaim responsibility for any injury to people or property resulting from any ideas, methods, instructions or products referred to in the content. ±
